# Low-Intervention Boundary-Risk Graph Calibration for Cross-Domain Few-Shot Hyperspectral Image Classification

**DOI:** 10.3390/s26154903

**Published:** 2026-08-03

**Authors:** Yuzhen Zhang, Yuanxiang Fan, Wenlong Wang

**Affiliations:** School of Computer Science and Technology, Kashi University, Kashgar 844000, China; zylooona@gmail.com (Y.Z.); userbro40@gmail.com (Y.F.)

**Keywords:** hyperspectral sensors, hyperspectral image classification, cross-domain few-shot learning, graph label propagation, boundary-risk calibration

## Abstract

Hyperspectral sensors provide high-dimensional spectral–spatial observations for land-cover analysis, but reliable classification remains difficult when only a few target-scene labels are available. Cross-domain few-shot hyperspectral image classification usually depends on sparse support samples, so final decisions can be unstable in low-margin regions where repairable errors and correctly classified long-tail samples are entangled. We propose Boundary-Risk Graph Calibration (BRGC), a risk-controlled calibration framework that improves support-set decision reliability. BRGC combines boundary-aware mixability training with inference-time graph residual calibration. During inference, support labels are clamped, an unlabeled target-query graph provides structural smoothing evidence, and the original classification scores are modified only through low-margin gating and bounded residual updates. On 10 target datasets with 10 random seeds, BRGC consistently improves its base classifier and achieves the highest macro-average OA, AA, and Kappa among matched-protocol transductive baselines. Repair/damage diagnostics, ablation studies, parameter sensitivity analysis, and cross-method adaptation show that BRGC improves scarce-label hyperspectral image interpretation by converting query-graph structure into low-intervention reliability evidence.

## 1. Introduction

Hyperspectral sensors record reflectance information over contiguous narrow spectral bands and provide both rich spectral signatures and spatial context for sensor-captured Earth observation imagery. Hyperspectral images (HSIs) have therefore been widely used in agricultural monitoring, urban material recognition, mineral exploration, and environmental observation [1,2,3]. Compared with RGB images, they can distinguish land-cover categories with subtle spectral differences. This sensing advantage also introduces high spectral dimensionality, inter-class similarity, spatial heterogeneity, and high annotation cost, which make hyperspectral image classification persistently challenging under limited labels and cross-scene transfer [3,4,5,6].

To improve hyperspectral image classification, previous studies have evolved from traditional machine learning and hand-crafted spectral–spatial features to deep neural networks. Support vector machines can build relatively robust decision boundaries in high-dimensional spectral spaces [2]. Spectral–spatial feature fusion, convolutional neural networks, residual networks, and Transformers further enhance local spatial modeling and long-range spectral dependency learning [3,4,5,6,7,8,9,10]. However, these methods usually assume that sufficiently many labeled samples are available in the target scene, whereas target-domain annotation is often expensive and time-consuming in remote sensor applications [4,5,6]. Few-shot learning and its HSI extensions have therefore become important, with the goal of recognizing target classes when only a few labeled samples are available for each class [11,12,13,14,15,16,17,18,19]. From a sensor-system perspective, the key issue is not only maximizing classification accuracy but also limiting unsafe corrections when scarce labels provide incomplete coverage of the target sensor scene.

In a more practical cross-domain few-shot HSI setting, a model first learns transferable representations from a source domain and then classifies target-domain queries using only a few support samples per target class. Existing methods improve cross-domain adaptation from multiple perspectives, including domain-transferable feature learning, graph relation aggregation, feature disentanglement, semantic prototypes, hierarchical prototype alignment, frequency alignment, and patch mixing [20,21,22,23,24,25,26,27,28,29]. These studies have substantially advanced cross-domain few-shot HSI classification. Nevertheless, most of them still make final predictions according to distances, relations, or prototype similarities between target support and query samples [12,16,17,18,23,24,25,26,27]. Thus, even when the representation network is strengthened, the final decision is still constrained by the coverage of the target support set.

Insufficient support coverage is particularly severe in the 5-shot regime. Prototypical networks and their HSI variants usually assume that a few support samples can approximate the target class distribution [12,16,17,18]. In cross-domain HSI, however, one class may contain multiple spectral–spatial sub-modes, while five support samples may cover only part of them. When a query sample comes from an uncovered long-tail sub-mode, it may be far from same-class support samples in the feature space while still belonging to the correct class. At the same time, crop subtypes, urban materials, shadows, bare soil, and vegetation boundaries often have similar spectral or textural responses, making correct boundary samples close to competing classes [3,4,5,6,24,25,26,27,28,29]. Therefore, low confidence, low classification margin, or a large support distance do not necessarily imply that a prediction is wrong.

This phenomenon makes hard-sample learning risky in cross-domain few-shot HSI classification. Mixup, CutMix, and attention-guided variants have shown that mixed or boundary samples can improve robustness to complex decision boundaries [30,31,32]. APMN also uses query patch mixing to strengthen local boundary representations for few-shot HSI classification [26]. However, hardness only indicates that a sample lies in an unstable region; it does not specify whether the sample should be corrected. Low-margin regions contain both repairable erroneous samples and high-risk samples that are already correctly classified but close to the boundary. If a method intervenes strongly based only on hardness, it may repair some erroneous samples while damaging originally correct boundary samples.

Graph learning and transductive inference provide another way to exploit an unlabeled target-domain structure. Semi-supervised graph propagation methods spread label information through local sample consistency and have been widely studied in HSI classification and few-shot learning [33,34,35,36,37,38,39,40,41,42]. Directly replacing the original classifier with graph propagation, however, is also risky. When samples with similar features but different semantics are connected by erroneous graph edges, propagation can amplify local errors. We therefore summarize a key residual bottleneck in cross-domain 5-shot HSI as boundary-risk entanglement: insufficient support coverage, inter-class similarity, and cross-domain shift jointly create a mixed boundary region where repairable wrong samples and high-risk correct samples have similar distances, margins, and confidence patterns.

Based on this observation, we propose Boundary-Risk Graph Calibration (BRGC) to improve decision reliability at the support-set interface of cross-domain few-shot hyperspectral image classifiers. BRGC uses boundary-aware mixability training to expose conflicts between low-margin queries and their competing classes. During inference, it builds a graph over unlabeled target queries, obtains smoothed scores through support-clamped graph propagation, and corrects the original classification scores only through low-margin gating and bounded residual updates. In this way, BRGC converts query-set structure into risk-controlled calibration evidence that complements sparse support samples while avoiding excessive intervention on stable predictions.

The main contributions of this paper are twofold.

1. We define boundary-risk entanglement as a decision-level reliability problem in cross-domain few-shot HSI, where low-margin regions contain both repairable erroneous samples and high-risk correct samples.
2. We propose BRGC, a risk-controlled boundary graph calibration framework for support-set decision-based cross-domain few-shot classifiers. It preserves the original base-classifier scores, converts support-clamped target query graph propagation into a bounded residual signal, and applies this signal only to low-margin candidate samples.

Extensive experiments on 10 target datasets with 10 random seeds, matched-protocol transductive baselines, component ablations, repair/damage diagnostics, parameter sensitivity analysis, and cross-base adaptation experiments are provided to validate the effectiveness and applicability boundary of BRGC.

The rest of this paper is organized as follows. Section 2 reviews related work. Section 3 presents the problem definition and preliminary analysis. Section 4 introduces the proposed BRGC framework. Section 5 reports experimental results and diagnostic analyses. Section 6 discusses the method boundary. Section 7 concludes this paper.

## 2. Related Work

HSI classification methods can broadly be divided into traditional spectral–spatial modeling methods and deep representation learning methods. Traditional approaches, represented by support vector machines and hand-crafted spectral–spatial features, can establish relatively robust decision boundaries in high-dimensional spectral spaces [2,3]. With the development of deep learning, convolutional networks, residual networks, and Transformers have been used to learn more discriminative features from local spatial neighborhoods and contiguous spectral bands [4,5,6,7,8,9,10]. Recent HSI-specific architectures further emphasize that spectral and spatial information should be modeled as a coupled data structure rather than as loosely fused branches. OSICN progressively compensates candidate spectral signatures during spatial feature extraction and uses a multi-branch network to reduce over-smoothing under limited training samples [43]. LGFormer directly processes original spectral–spatial patches and combines 3D convolutional local attention with Transformer-style global attention, thereby preserving spectral–spatial correlation while capturing both local details and long-range context [44]. These methods perform well when sufficient labels are available, but their performance usually depends on many labeled samples in the target scene. When only a few labels are available for each class, deep models are more vulnerable to overfitting, class imbalance, and spatial heterogeneity [4,5,6].

Few-shot learning has been introduced into HSI classification to reduce the dependence on target-domain labels. Early few-shot HSI methods often borrowed metric-learning ideas from prototypical networks, relation networks, and Siamese networks and classified queries through distances or relations between support and query samples [11,12,13,14,15,16,17,18,19]. These methods naturally fit low-label protocols such as 5-shot evaluation. Their core assumption, however, is that a few support samples can adequately represent the target class distribution. When a class contains multiple spectral–spatial sub-modes, the limited representativeness of support samples directly affects the final decision boundary. This is the decision-level problem studied in this paper.

Cross-domain few-shot HSI classification further considers the distribution gap between source and target domains. Existing methods can roughly be grouped into three categories. The first learns transferable features, such as DCFSL, which uses cross-domain episodic learning, and FDFSL, which uses feature disentanglement to reduce domain-related factors [20,23]. The second emphasizes class relations or prototype alignment, such as Gia-CFSL with graph information aggregation, semantic-guided prototype learning, and MLPA with multi-level prototype alignment [21,24,25]. The third introduces richer auxiliary evidence, including class-wise attention, frequency alignment, topological information, and patch mixing, to improve class separability and local robustness under cross-domain conditions [22,26,27,28,29].

Although these methods mitigate domain shift from different perspectives, most still share a similar support-set decision interface during target-domain evaluation. Given target support and query samples, the model classifies a query according to nearest support samples, class prototypes, or support-query relations [16,17,18,20,23,24,25,26,27]. Therefore, cross-domain few-shot HSI classification is not only a representation transfer problem. It is also a question of whether a small target support set can cover intra-class variation. BRGC addresses this decision interface by adding risk-controlled graph residual calibration to the base support-set classifier.

Another related direction is hard-sample learning and boundary enhancement. Hard-sample learning exposes models to low-confidence samples, class-boundary samples, or mixed samples to improve robustness to complex decision boundaries. Mixup smooths the decision boundary through linear interpolation of inputs and labels, CutMix constructs local mixed samples through regional replacement, and TransMix uses attention to assign mixed labels [30,31,32]. In few-shot HSI classification, APMN combines patch mixing with attention and uses query-only mixing to improve robustness to complex local patches [26]. These methods show that boundary and mixed samples are valuable for improving few-shot discriminability.

However, hard-sample enhancement usually focuses on which samples are difficult, rather than which difficult samples are safe to intervene on. In cross-domain 5-shot HSI, a low-margin sample may be an erroneous sample that can be repaired, or it may be a correctly predicted long-tail sample that is far from the support set. If training or inference updates are driven only by hardness, the model may repair erroneous boundary samples while damaging correct boundary samples. BRGC therefore treats hard samples as boundary-risk signals. It uses them to expose class conflicts during training and then limits inference-time intervention through low-margin gating and residual clipping.

Graph learning and semi-supervised learning provide a way to exploit unlabeled sample structure under limited labels. Classical graph label propagation is usually based on local–global consistency or harmonic-function assumptions, where neighboring samples and samples on the same manifold are likely to share labels [34,35]. In HSI classification, graph methods have been used to model spectral–spatial similarities among pixels, superpixels, or local regions, and to alleviate label scarcity through label propagation, random walks, or multi-feature graph learning [33,36,37,38,39]. For multimodal remote sensing classification, THSGR extends graph-based modeling to HSI-SAR/LiDAR data by constructing heterogeneously salient graph representations across modalities and by replacing standard self-attention with a multi-convolutional modulator for more efficient long-range dependency modeling [45]. These studies indicate that target-domain sample relations can provide useful constraints beyond a small labeled set.

Transductive few-shot learning also uses query-set structure to calibrate decision boundaries. TIM optimizes transductive inference through information maximization on fixed features. Prototype rectification adjusts class prototypes using unlabeled queries. protoLP iteratively refines predictions through prototypes and graph label propagation [40,41,42]. These methods and BRGC all recognize the value of unlabeled query structure. The difference is that BRGC targets high-risk boundary regions in cross-domain HSI. It does not directly replace the base classifier with graph propagation. Instead, it fixes the support labels, uses the difference between graph-propagated scores and original scores as a bounded residual, and applies this residual only to low-margin queries. Thus, to combine the stability of support-set decisions with the complementarity of target query graph structure, BRGC seeks a low-intervention transductive calibration strategy that corrects only a small number of unstable boundary predictions while preserving the primary judgment of the base classifier.

BRGC is therefore complementary to recent HSI representation methods and graph-based remote sensing methods. OSICN and LGFormer mainly redesign the feature extractor so that spectral–spatial information is fused earlier and represented more effectively. THSGR uses graph representation as a multimodal feature-learning mechanism for HSI-SAR/LiDAR classification. In contrast, BRGC does not introduce a new spectral–spatial backbone or multimodal fusion model. It operates after the support-set classifier has produced its original scores, keeps these base scores as the primary evidence, and uses a support-clamped query graph only as a low-margin residual calibration signal. This distinction is important because the main problem addressed here is not how to learn a stronger full-supervision representation but how to reduce decision risk when a cross-domain few-shot classifier must rely on only a few labeled target support samples.

To avoid interpreting BRGC as an APMN post-processing step or standard graph label propagation, Table 1 summarizes the mechanism-level differences between BRGC and related methods. APMN mainly improves boundary representations through query patch mixing, but its inference stage still relies on target support-set decisions. Standard Graph LP, TIM, and protoLP use a query-set structure but typically use graph propagation, transductive objectives, or prototype rectification as new final decision sources. BRGC differs by imposing three low-intervention constraints: it preserves the original base-classifier scores as the main decision source, clamps support-sample labels so that the target query graph only provides structural smoothing information, and uses only the difference between graph-propagated scores and original scores as a gated and clipped residual update. Therefore, BRGC is not merely “APMN plus graph propagation” but a residual transductive calibration framework for boundary-risk entanglement.

## 3. Problem Definition and Preliminary Analysis

This section formalizes the problem studied in this paper and explains why low-margin hard samples in cross-domain few-shot HSI should not be treated uniformly as repairable errors. Let the source domain be $D_{s}=\left\{\left(x_{i}^{s},y_{i}^{s}\right)\right\}$ and the target domain be $D_{t}$. In cross-domain few-shot learning, a model first learns a feature extractor or classification rule from the source data and then receives only *K* labeled support samples per class in a target episode. This paper focuses on the 5-shot setting with K=5. Given a target support set

(1) $$S={\left\{\left(x_{i},y_{i}\right)\right\}}_{i=1}^{N_{s}}$$

and a target query set

(2) $$Q={\left\{x_{j}\right\}}_{j=1}^{N_{q}},$$

the model predicts the label of each query sample $x_{j}$. Let the target class set be $C_{t}=\left\{1,\ldots ,C_{t}\right\}$. Many cross-domain few-shot HSI methods have different training objectives and network architectures but can be abstracted as support-set decision-based classifiers during target-domain evaluation. Given a feature extractor ϕ(·), the original similarity score of a query sample *q* for class $c\in C_{t}$ can be written as

(3) $$s_{c}\left(q\right)=h\left(\phi \left(q\right),\Phi \left(S_{c}\right)\right),$$

where $S_{c}$ denotes the support samples of class *c*, $\Phi (S_{c})$ denotes the support features or class prototype, and h(·) is a support-query scoring function for which a larger value indicates higher class compatibility. If a base method outputs distances, such as nearest-support distances, we use the negative distance or a monotonic decreasing transform to convert it into a similarity score. The final prediction is

(4) $$\hat{y}\left(q\right)=\arg\max_{c\in C_{t}}s_{c}\left(q\right).$$

This formulation is interpretable and suitable for few-shot evaluation. It also explicitly ties the target-domain classification quality to the representativeness of a few support samples. In other words, even when ϕ(·) extracts discriminative features, the final score $s_{c}\left(q\right)$ may still be biased because $S_{c}$ insufficiently covers the target class.

To describe the instability of support-set decisions, we use two quantities that are directly related to the proposed method. The first is the distance from a query to the support evidence of a class. With a nearest-support formulation, it can be defined as

(5) $$d_{c}\left(q\right)=\min_{\left(x_{i},y_{i}\right)\in S_{c}}{∥\phi \left(q\right)-\phi \left(x_{i}\right)∥}_{2}.$$

For prototype- or relation-based classifiers, $d_{c}\left(q\right)$ can also be interpreted as a generalized dissimilarity between the query and the support evidence of class *c*. A large $d_{\hat{y}}\left(q\right)$ indicates that, although the query is assigned to class $\hat{y}$, it is far from the support samples of that class in the feature space. Such a prediction relies more heavily on extrapolation.

The second quantity is the classification margin. Let $c_{1}$ and $c_{2}$ denote the classes with the highest and second-highest scores:

(6) $$c_{1}\left(q\right)=\arg\max_{c\in C_{t}}s_{c}\left(q\right),\;c_{2}\left(q\right)=\arg\max_{c\in C_{t},\;c\neq c_{1}\left(q\right)}s_{c}\left(q\right).$$

The classification margin is defined as

(7) $$m\left(q\right)=s_{c_{1}\left(q\right)}\left(q\right)-s_{c_{2}\left(q\right)}\left(q\right).$$

A small m(q) value indicates that the model has no clear advantage for the predicted class $c_{1}$ over the competing class $c_{2}$, and the query lies in an unstable decision region. Conventional hard-sample mining often prioritizes such low-margin samples. In cross-domain 5-shot HSI, however, a low margin only indicates prediction instability. It does not mean that intervention will necessarily improve the prediction.

Combining support distance and classification margin yields a finer boundary region. Let $\tau_{m}$ and $\tau_{d}$ be the margin threshold and support-distance threshold. The set of low-margin queries that are far from support evidence can be written as

(8) $$B=\{q\in Q|m\left(q\right)\leq \tau_{m},\;d_{c_{1}\left(q\right)}\left(q\right)\geq \tau_{d}\}.$$

The set B is the high-risk boundary region considered in this paper. If ground-truth labels y(q) are used for offline diagnosis, B can be further divided into two subsets:

(9) $$B_{wrong}=\left\{q\in B|c_{1}\left(q\right)\neq y\left(q\right)\right\},$$

and

(10) $$B_{risky}=\left\{q\in B|c_{1}\left(q\right)=y\left(q\right)\right\}.$$

The first subset corresponds to hard samples that are far from the support set and incorrectly predicted, and it can be regarded as potentially repairable. The second corresponds to samples that are far from the support set but correctly predicted, suggesting that they may come from long-tail sub-modes of the correct class that are not covered by the 5-shot support set. During unlabeled inference, both subsets exhibit low margins, large support distances, or low confidence. They are therefore difficult to distinguish reliably using only the current score state.

We call this phenomenon boundary-risk entanglement. Formally, the high-risk boundary region can be viewed as a disjoint union:

(11) $$B=B_{wrong}\dot{\cup }B_{risky},\;B_{wrong}\cap B_{risky}=⌀,$$

where $B_{wrong}$ contains repairable erroneous samples and $B_{risky}$ contains high-risk correct samples.

It is important to note that $B_{wrong}$ and $B_{risky}$ are used only for offline diagnosis and problem interpretation. Ground-truth labels are unavailable during formal inference. Therefore, if a method forcibly corrects all low-margin or support-far queries, it may repair errors in $B_{wrong}$ while damaging originally correct samples in $B_{risky}$.

Our preliminary analysis indicates that boundary-risk entanglement is jointly caused by three factors. First, a 5-shot support set covers only a small part of the target class distribution. When the true class contains multiple sub-modes, a few support samples may cover only some of them. Second, different crops, urban materials, shadows, bare soil, and other HSI categories often have similar spectral responses or spatial textures, naturally placing correct boundary samples close to competing classes. Third, cross-domain shift makes the feature distance learned from the source domain not fully equivalent to the semantic distance in the target domain, further amplifying the uncertainty caused by support coverage deficiency.

These factors suggest that the residual errors of cross-domain few-shot HSI are not only due to insufficient feature extraction but also to over-reliance on sparse support evidence during target-domain decision making. More importantly, unlabeled proxies such as low margin, low confidence, support distance, augmentation consistency, and support-subset stability mainly describe whether a sample is currently difficult. They do not directly determine whether an intervention will repair an error or damage a correct prediction. Therefore, this paper uses support distance mainly for problem diagnosis and offline analysis, not as a sufficient condition for separating $B_{wrong}$ from $B_{risky}$ during formal inference. The deployed BRGC version adopts a more conservative low-margin gate, treats low-margin samples only as candidates, and reduces damage risk through residual magnitude constraints. In other words, BRGC does not claim to precisely identify, without labels, which samples should or should not be corrected. Instead, it satisfies three weaker but executable constraints: the original support-set classifier should remain the primary decision source, calibration should preferentially act on low-margin boundary regions, and the update magnitude should be bounded to reduce damage to $B_{risky}$. BRGC is built under this problem definition as a low-intervention graph residual calibration framework.

## 4. Proposed Method: Boundary-Risk Graph Calibration

**Figure 1:**
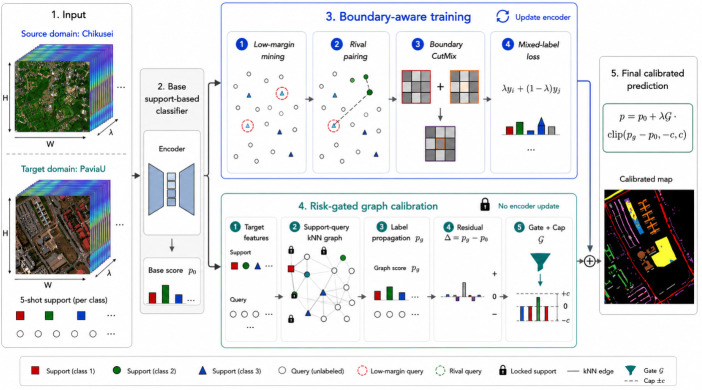
Main framework of Boundary-Risk Graph Calibration (BRGC). The workflow proceeds from left to right. Source-domain hyperspectral image (HSI) data are first used for episodic training, where boundary-aware mixability training exposes low-margin class conflicts. In a target-domain 5-shot episode, the base classifier obtains original query scores from the target support set. BRGC then builds a query graph over target support and unlabeled query samples, performs support-clamped graph propagation, and uses only the difference between graph-propagated scores and original scores as a residual signal. The low-margin gate determines which queries are eligible for calibration, residual clipping limits the update magnitude, and the final output is a calibrated prediction map. The figure highlights the key role of BRGC: the base representation and original prediction remain the primary decision source, while query-graph evidence is introduced as a risk-controlled low-intervention calibration signal. Gray cells in the Boundary CutMix schematic denote patch regions selected for replacement or mixing.

BRGC is a low-intervention calibration framework for support-set decision-based cross-domain few-shot HSI classifiers. It operates at the support-set decision interface and imposes training- and inference-stage constraints around the boundary-risk entanglement defined in Section 3. During training, BRGC constructs boundary-mixed samples from low-margin queries and competing-class samples, exposing the model to potentially confusing regions. During inference, BRGC builds a graph over target support samples and unlabeled query samples, and it performs support-clamped graph propagation. The graph-propagated scores are then converted into a bounded residual update, which provides a small correction through low-margin gating and residual clipping. The overall workflow is shown in Figure 1.

### 4.1. Overall Framework

Let the base few-shot classifier be $F_{\theta }$. BRGC only requires $F_{\theta }$ to provide three outputs: support features, query features, and original class scores for the queries. Thus, the method interface of BRGC is not tied to a specific network layer but to the support-set decision interface consisting of support features, query features, and original scores. As long as a base method can produce these quantities, BRGC can be attached as a training strategy and inference calibration module. In the main instantiation, $F_{\theta }$ uses a patch-based spectral–spatial encoder to extract local HSI representations and computes original query class scores from the target support samples.

Given a query sample *q*, the base classifier first produces class scores s(q), which are normalized into the original probability distribution:

(12) $$p_{0}\left(q\right)=\mathrm{Softmax}\left(s\left(q\right)\right).$$

BRGC then obtains a graph-propagated probability $p_{g}\left(q\right)$ from the target query graph. The final prediction score is

(13) $$p_{\mathrm{final}}\left(q\right)=\mathrm{Norm}\left(p_{0}\left(q\right)+\lambda G\left(q\right)\cdot \mathrm{clip}\left(p_{g}\left(q\right)-p_{0}\left(q\right),-c,c\right)\right),$$

where G(q)∈{0,1} is the low-margin gate, λ controls the calibration strength, *c* is the residual clipping threshold, and Norm(·) denotes sample-wise normalization after non-negative clipping. More precisely, for a vector $z\in R^{C_{t}}$, BRGC uses the ϵ-smoothed normalization

(14) $${\left[\mathrm{Norm}\left(z\right)\right]}_{c}=\frac{\max(z_{c},0)+\epsilon }{\sum_{r\in C_{t}}\max\left(z_{r},0\right)+C_{t}\epsilon },$$

where $\epsilon =10^{-12}$ in the implementation and is used only as a numerical safeguard to avoid degenerate all-zero normalization; it is not a tuned hyperparameter. This formulation reflects the main principle of BRGC: the original support-set classifier remains the primary decision source, while the target query graph only provides bounded residual information.

### 4.2. Boundary-Aware Mixability Training

Boundary-aware mixability training exposes the low-margin boundary regions described in Section 3. BRGC first trains the base classifier using a standard episodic few-shot procedure and activates boundary mixing after warm-up. For each episode, the model computes the classification margins of all query samples and selects low-margin queries as boundary anchors. Given an anchor sample $q_{a}$ with label $y_{a}$, the model determines the strongest competing class $y_{b}$ from the current class scores and selects a feature-neighbor query $q_{b}$ from that class. If no suitable competing-class sample exists in the episode, the pairing falls back to a random sample from a different class.

In the main instantiation, the boundary mixing module follows the query patch mixing form in APMN that is compatible with HSI patch representations, including the patch-based encoder, query-only mixing, and attention-guided local replacement. The new role of BRGC is not to reinvent this mixing operator, but to position it as the boundary exposure stage within a boundary-risk calibration framework. During training, low-margin queries expose potential class conflicts. During inference, the target query graph and bounded residual decide whether a small correction should be applied. Therefore, APMN provides a boundary representation enhancement basis, whereas BRGC further introduces target-unlabeled graph structure, original score preservation, low-margin candidate gating, and residual clipping calibration.

After a sample pair is selected, BRGC constructs a boundary-mixed sample $\tilde{x}$ using attention-guided CutMix. In the main implementation, this module adopts a query patch mixing form compatible with patch-based HSI few-shot classifiers. For other support-set decision-based base methods, it can be replaced by another boundary mixing strategy that follows the same “low-margin sample versus competing class” principle. The mask region or attention weights determine two non-negative mixing weights, $\omega_{a},\omega_{b}\in \left[0,1\right]$ with $\omega_{a}+\omega_{b}=1$, for the anchor label $y_{a}$ and the competing label $y_{b}$. The mixed training loss is

(15) $$L_{mix}=\omega_{a}\mathrm{CE}\left(F_{\theta }\left(\tilde{x}\right),y_{a}\right)+\omega_{b}\mathrm{CE}\left(F_{\theta }\left(\tilde{x}\right),y_{b}\right).$$

Here, CE(·,·) denotes the cross-entropy loss and $F_{\theta }$ denotes the base classifier with parameters θ. The purpose of this module is not to increase training difficulty indiscriminately but to amplify conflicts between low-margin samples and competing classes. The model can therefore observe boundary regions more frequently during training and provide a more stable feature basis for subsequent low-margin calibration.

### 4.3. Support-Clamped Target Query Graph Construction

During inference, BRGC uses the trained $F_{\theta }$ to extract target support and query features. All support and query samples form the graph node set:

(16) V=S∪Q.

A *k*-nearest-neighbor graph is constructed in the feature space. For nodes *i* and *j*, if *j* belongs to the neighbor set of *i*, the edge weight is

(17) $$W_{ij}=\exp\left(-\frac{∥\phi \left(x_{i}\right)-\phi \left(x_{j}\right){∥}_{2}^{2}}{\tau }\right),$$

and otherwise $W_{ij}=0$. The graph is then symmetrized and row-normalized to obtain the transition matrix T. This graph captures local manifold relations between target support and query samples and provides unlabeled structural information for query regions not sufficiently covered by the few support samples. BRGC uses k=20 and temperature τ=0.05 by default.

### 4.4. Graph Propagation and Residual Calibration

Let $P^{\left(0\right)}$ be the initial class probability matrix. For support nodes, the initial distribution is the ground-truth one-hot label. For query nodes, the initial distribution is the base classifier output $p_{0}\left(q\right)$. Graph propagation is iterated as

(18) $$P^{\left(t+1\right)}=\alpha TP^{\left(t\right)}+\left(1-\alpha \right)P^{\left(0\right)}.$$

After each iteration, the support nodes are clamped back to their ground-truth labels to prevent support anchors from drifting during propagation:

(19) $$P_{S}^{\left(t+1\right)}=Y_{S},$$

where $Y_{S}$ is the one-hot label matrix of the target support samples. After $L_{g}$ iterations, the graph-propagated query probability $p_{g}\left(q\right)$ is obtained. BRGC uses α=0.8 and $L_{g}=30$ by default.

Unlike standard graph label propagation, BRGC does not directly use $p_{g}\left(q\right)$ as the final prediction. It only uses the change induced by graph propagation:

(20) $$\Delta \left(q\right)=p_{g}\left(q\right)-p_{0}\left(q\right).$$

To avoid overly strong updates caused by graph noise, BRGC clips Δ(q) element-wise:

(21) $$\bar{\Delta }\left(q\right)=\mathrm{clip}\left(\Delta \left(q\right),-c,c\right).$$

The update is applied only to low-margin samples:

(22) $$p_{\mathrm{final}}\left(q\right)=\mathrm{Norm}\left(p_{0}\left(q\right)+\lambda G\left(q\right)\bar{\Delta }\left(q\right)\right).$$

Here, G(q)=1 means that *q* belongs to the low-margin candidate set, and G(q)=0 means that the original prediction is kept unchanged. By default, BRGC selects the one-third of query samples with the lowest margins as calibration candidates and uses λ=0.8 and c=0.1. Ground-truth labels are not used, and support distance is not used to decide whether a sample must belong to $B_{wrong}$ or $B_{risky}$. The low-margin gate only provides a conservative candidate set, while the actual update is jointly limited by the graph residual direction and clipping magnitude. This design constrains graph propagation to a bounded residual term, making it closer to risk-controlled decision-level calibration than to an unconstrained transductive classifier.

At the implementation level, this design also differs from standard Graph LP, TIM, and protoLP. Standard Graph LP tends to use the propagated label distribution as the final classification basis. TIM and prototype rectification methods usually re-optimize the entire query prediction set through a transductive objective or prototype movement. protoLP iteratively updates prototypes and graph propagation relations. BRGC does not move support labels, does not re-estimate base-classifier parameters, and does not allow graph propagation to overwrite all query scores. It only computes the residual $p_{g}\left(q\right)-p_{0}\left(q\right)$ and controls the residual magnitude by λ and *c* within the low-margin candidate set. Therefore, the technical goal of BRGC is not to construct a stand-alone transductive classifier but to add a switchable, clipped, and interpretable graph-structure calibration term to an existing support-set decision result.

### 4.5. Protocol Boundary

BRGC uses unlabeled target query features to construct a graph during inference and is therefore a target-unlabeled transductive few-shot method. It does not use query labels for gating, graph propagation, or parameter selection, but it does use unlabeled structural information among query samples. Experimental comparisons must therefore explicitly state the protocol difference. BRGC should not be described as a strict inductive few-shot method.

Algorithm 1 summarizes the main procedure of BRGC.
**Algorithm 1** Boundary-Risk Graph Calibration (BRGC)Input: source-domain training set, target support set S, target query set Q, base few-shot classifier F_theta Output: predicted labels for target query samples
1.  Train the base classifier F_theta with episodic training2.  After warm-up, compute query classification margins within each episode3.  Select low-margin query samples as boundary anchors4.  For each anchor, find a neighboring query from the strongest competing class5.  Construct boundary-mixed samples and optimize F_theta with the weighted mixed-sample loss6.  After training, extract target support and target query features7.  Compute original class probabilities p0(q) from the support samples8.  Build a kNN graph over support and query samples9.  Clamp support labels and perform target query graph propagation to obtain pg(q)10.Apply bounded residual updates to low-margin query samples11.Output the final prediction pfinal(q)


## 5. Experimental Results and Analysis

### 5.1. Datasets, Protocol, and Evidence Design

We use the public Chikusei hyperspectral dataset as the unified source-domain training dataset and evaluate BRGC on ten target HSI datasets. Chikusei was collected by the Headwall Hyperspec-VNIR-C sensor, has a spatial size of 2517×2335, a spatial resolution of approximately 2.5 m, 128 spectral bands, and 19 land-cover classes. Its categories include water, bare soil, natural vegetation, agricultural weeds, forest, grassland, rice at different growth stages, row crops, plastic greenhouses, man-made objects, asphalt, and paved ground. Previous cross-domain few-shot HSI studies commonly use Chikusei as the source domain and IndianPines, PaviaU, Salinas, and other datasets as target domains [20,21,26]. We follow this source-domain setting and extend the target domains to IndianPines, Salinas, PaviaC, PaviaU, KSC, Botswana, Houston13, WHUHiLongKou, WHUHiHanChuan, and WHUHiHongHu, covering classic agricultural scenes, urban material scenes, wetland vegetation scenes, and modern UAV high-resolution crop scenes.

All experiments use the Chikusei → target cross-domain 5-shot setting. During training, episodic tasks are constructed from the Chikusei source domain to learn transferable spectral–spatial representations. In the target domain, only 5 labeled support samples per class participate in target support-set decisions, and the remaining labeled samples are used only for offline testing. When the number of target-domain classes exceeds the 19 source-domain classes in Chikusei, source episodes are constructed from available source classes, whereas target episodes, final support-set decisions, and class-wise evaluation keep the full class set of the target dataset. Each target dataset is evaluated with 10 random seeds, and the base classifier is trained for 5000 episodes. The evaluation metrics are overall accuracy (OA), average accuracy (AA), and the Kappa coefficient. We report the mean and standard deviation over 10 random seeds.

BRGC uses target support samples and unlabeled query samples to build a graph at inference time, but it does not use query labels. We therefore explicitly mark it as a target-unlabeled transductive 5-shot calibration protocol. DCFSL, Gia-CFSL, FDFSL, MLPA, and APMN are reported as strict inductive cross-domain few-shot baselines. Target-domain 5-shot methods such as SVM, 3DCNN, and SSRN are used only as protocol-stratified references [2,8,46]. To test whether BRGC is merely a standard query-graph post-processing method, we also implement four matched-protocol transductive baselines on the same APMN features, target support sets, and unlabeled query sets: Graph LP, LaplacianShot-style update, TIM-style adaptation, and iLPC-style iterative propagation. Thus, the key comparisons address three questions: whether BRGC consistently improves its base support-set classifier, whether it is competitive with standard transductive graph calibration, and whether its gains follow the low-intervention repair/damage mechanism.

The experiments were implemented in Python 3.10 using PyTorch 2.5.1 with CUDA 11.8 support. The main environment also used NumPy (version 1.26 or later), SciPy (version 1.11 or later), scikit-learn (version 1.4 or later), h5py (version 3.10 or later), spectral (version 0.23 or later), and Matplotlib (version 3.8 or later). The runs were executed on a workstation equipped with four NVIDIA A40 GPUs, each with 46,068 MiB memory, using driver version 570.124.06. The dependency specification is maintained in the project pyproject.toml and uv.lock files. Source code, configuration files, evaluation scripts, and processed result summaries will be released in a public repository after acceptance; before release, they are available from the corresponding author upon reasonable request for review and reproducibility checks.

To avoid misinterpreting different protocols as fully homogeneous comparisons, we explicitly record the information available to each method type. Table 2 summarizes the experimental protocols. BRGC can access unlabeled target query features during inference to build a query graph and perform graph residual calibration, but query ground-truth labels are used only for offline evaluation and not for training, gating, propagation, or parameter selection.

Although all matched-protocol transductive methods use unlabeled target query features, their intervention forms and assumptions differ. Table 3 lists their decision mechanisms in our implementation. Graph LP and LaplacianShot emphasize query-graph smoothing. TIM emphasizes query-set entropy and class-marginal adaptation. iLPC relies on iterative pseudo-label propagation. BRGC preserves base scores as the main decision source and uses only the difference between graph-propagated and base scores as a gated and clipped residual. The main difference is therefore not whether the query graph is used, but how its intervention on original support-set decisions is constrained.

The experiments are organized around three evidence levels: overall effectiveness, mechanism explanation, and applicability boundary. Table 4 and Table 5 report the main performance comparisons. Spatial visualizations and class-level analyses examine boundary confusion and repair behavior. Ablation and repair/damage diagnostics test the low-intervention design. Cross-base adaptation, parameter sensitivity, and paired-seed tests evaluate applicability and robustness. To keep the main text focused, only representative class-wise and visualization evidence is retained in the main manuscript, while complete class-wise, sensitivity, transductive-baseline, and cross-method results are placed in the Supplementary Materials.

**Figure 2:**
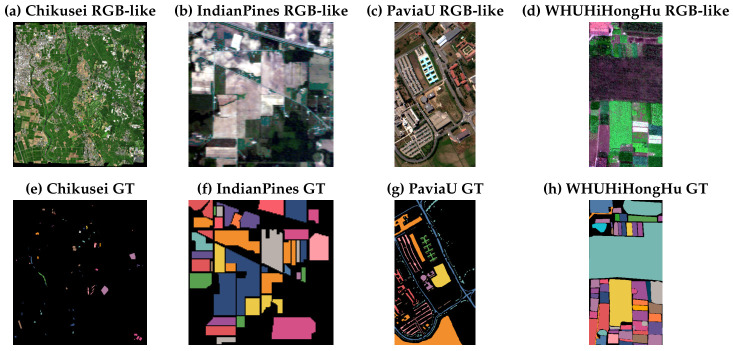
Approximate visible RGB composites and ground-truth maps of the source and representative target datasets. Panels (**a**–**d**) show RGB-like composites, and panels (**e**–**h**) show the corresponding ground-truth (GT) maps. Each column corresponds to one dataset. The RGB-like composites use bands close to visible red, green, and blue wavelengths, which visualize spatial textures, scene scale, and land-cover layout. The GT maps visualize class distributions, labeled regions, and class boundaries. Black denotes unlabeled or non-evaluated background regions. The RGB-like composites are used only for scene visualization and interpretation, not for model training or quantitative evaluation.

Figure 2 shows approximate visible-light RGB composites and ground-truth maps for the source domain and representative target domains. Because HSI datasets usually do not provide standard three-channel true-color images, we select bands close to visible red, green, and blue wavelengths and apply robust stretching to obtain RGB-like scene thumbnails. The ground-truth maps show class distributions, labeled regions, and class boundaries. Chikusei covers a relatively large urban-agricultural mixed scene, whereas IndianPines, PaviaU, and WHUHiHongHu represent small-scale crops, urban materials, and high-resolution crop scenes, respectively. This difference indicates that the experiments involve not only class differences but also substantial variation in spatial scale, imaging condition, and boundary complexity.

### 5.2. Overall Performance and Matched-Protocol Transductive Comparison

We first examine whether BRGC provides stable gains across multiple target domains. Table 4 summarizes OA comparisons among DCFSL, Gia-CFSL, FDFSL, MLPA, APMN, and BRGC on 10 target datasets. All results are reported as mean and standard deviation over 10 random seeds. Because BRGC is a target-unlabeled transductive calibration method as defined in Table 2, Table 4 should not be interpreted as a strict same-protocol comparison between transductive calibration and strict inductive methods. Instead, after making the available information explicit, it shows the practical effect of low-intervention graph calibration on top of a support-set decision classifier. The most direct comparison is with the base classifier APMN. Under the same target datasets, 5-shot setting, and random seeds, BRGC improves APMN on all 10 target datasets, indicating that its gain is not tied to a single target domain and remains positive across agricultural, urban material, wetland vegetation, and modern high-resolution crop scenes.

Macro-average results further support this trend. Table 5 reports both strict inductive baselines and matched-protocol target-unlabeled transductive baselines. Compared with APMN, BRGC improves macro OA from 89.308% to 90.032%, macro AA from 89.548% to 90.278%, and macro Kappa from 87.517 to 88.353. Graph LP and LaplacianShot also improve macro OA by approximately +0.58 on the same APMN features, indicating that the target query graph itself contains useful information. However, both rely directly on propagated scores as the main decision source and lack low-margin candidates, bounded residuals, and original-score preservation. BRGC obtains the highest macro OA, AA, and Kappa among these matched transductive baselines. These results indicate that the value of BRGC is to constrain query-graph evidence as low-intervention residual calibration while retaining competitive macro-average performance under the matched transductive protocol.

At the dataset level, BRGC shows clearer advantages on IndianPines, Houston13, WHUHiHanChuan, and WHUHiHongHu, which involve fine-grained crops, urban materials, or high-resolution complex scenes. These scenes are more likely to contain inter-class similarity, dense boundaries, and insufficient support coverage. The margins are smaller on KSC, PaviaC, Salinas, and WHUHiLongKou, suggesting that when the base classifier is already stable, the remaining calibration space is limited. Dataset-level comparisons with Graph LP and LaplacianShot reveal the same boundary. BRGC achieves the highest OA in the matched transductive group on 7 of 10 target datasets, whereas standard graph propagation is slightly better or comparable on PaviaU, Botswana, and KSC. Thus, graph residual gating is not superior to full graph propagation for every data distribution. Complete dataset-level transductive results are reported in Table S13.

**Figure 3:**
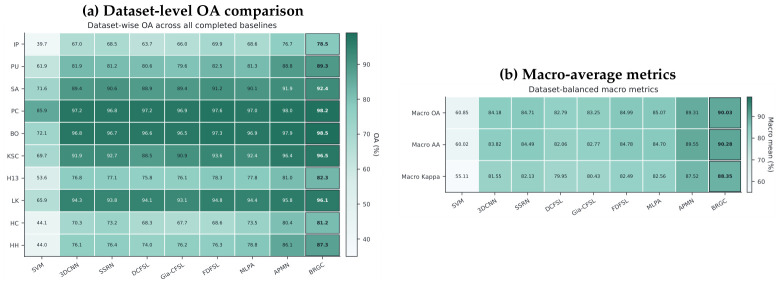
Overall performance comparison on target datasets. Panel (**a**) shows an OA heatmap of SVM, 3DCNN, SSRN, DCFSL, Gia-CFSL, FDFSL, MLPA, APMN, and BRGC on 10 target datasets. Panel (**b**) summarizes macro-average OA, AA, and Kappa. Darker colors indicate higher values, and black boxes mark the best result for each dataset or metric. This figure evaluates the stability of the overall gains obtained by BRGC. Within the information scope defined in Table 2, BRGC improves over its base method APMN on all target datasets and maintains the highest macro-average metrics among protocol-stratified references.

TIM-style adaptation is substantially lower than other matched transductive baselines under our protocol. This mainly reflects a mismatch between the query-set class-marginal assumption of TIM-like methods and the full-image HSI testing scenario: target query sets are usually long-tailed, whereas entropy and marginal adaptation in TIM tend to push predictions toward a more balanced distribution. We therefore retain TIM as a diagnostic baseline showing that not all transductive objectives are suitable for full-image few-shot HSI, rather than treating it as the strongest main comparison for BRGC. Comparisons with Graph LP, LaplacianShot, and iLPC better reflect the competitiveness of graph-structure calibration. Figure 3 summarizes the dataset-level OA distribution and macro-average metrics corresponding to the overall comparison.

### 5.3. Classification Maps and Spatial Correctness Analysis

Numerical tables show the average accuracy advantage of BRGC, but HSI classification also requires spatial maps to examine whether the method improves only a few pixels or improves spatially continuous regions, boundary regions, and confused classes. We visualize IndianPines, PaviaU, and WHUHiHongHu as three representative target datasets. IndianPines reflects fine-grained crop confusion, PaviaU reflects urban materials and shadow boundaries, and WHUHiHongHu reflects high-resolution fine-grained crop scenes.

**Figure 4:**
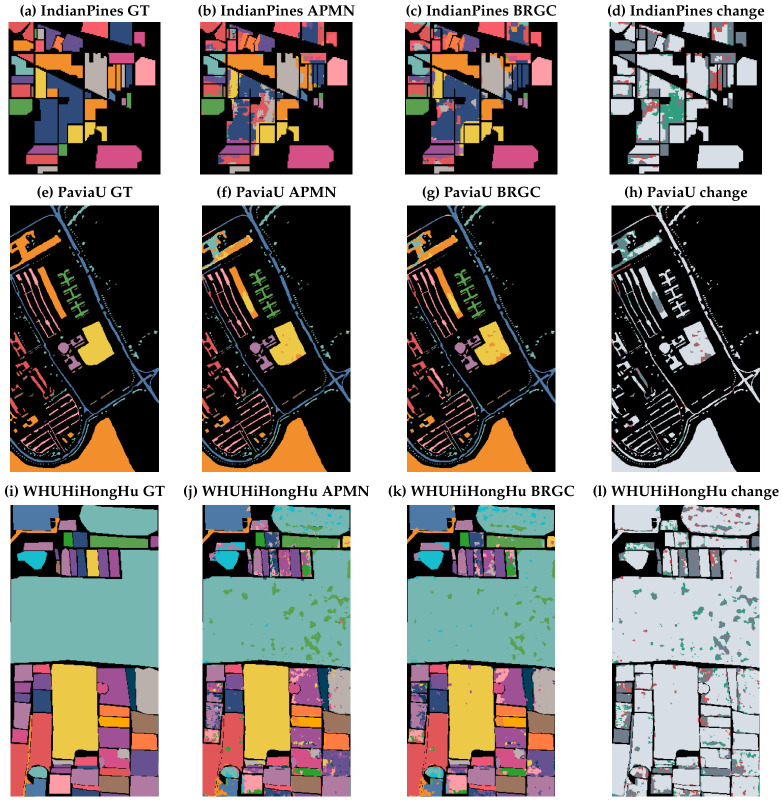
Classification maps and correctness-change maps for representative target datasets. Panels (**a**–**d**), (**e**–**h**), and (**i**–**l**) correspond to IndianPines, PaviaU, and WHUHiHongHu, respectively. Within each dataset, the four panels show the ground truth (GT), APMN classification map, BRGC classification map, and the error-change map of BRGC relative to APMN. Colors in the GT, APMN, and BRGC maps denote class labels and are used to compare spatial classification results. The change panels denote correctness changes rather than class labels: black represents unlabeled or non-evaluated background, light gray indicates pixels correctly predicted by both APMN and BRGC, dark gray indicates pixels incorrectly predicted by both methods, green indicates repaired pixels, and red indicates damaged pixels. This figure evaluates whether BRGC mainly performs local repairs in boundary and confused regions rather than substantially altering the classification map.

Figure 4 shows ground truth, APMN classification maps, BRGC classification maps, and error-change maps for the three datasets. The error-change maps mark pixels that are wrong under APMN but correct under BRGC as repaired, pixels that are correct under APMN but wrong under BRGC as damaged, and pixels that are correct or wrong under both methods as correct kept and error kept. BRGC does not substantially alter the classification map. Its changes concentrate around field boundaries, urban material boundaries, and fine-grained crop regions.

Finer local zoom-in maps are provided in Supplementary Figure S1. They examine whether BRGC corrections occur near field edges, urban material interfaces, and fine-grained crop patches. They are not repeated in the main text to avoid redundancy with the spatial evidence in Figure 4.

### 5.4. Confusion Matrix and Class-Level Change Analysis

**Figure 5:**
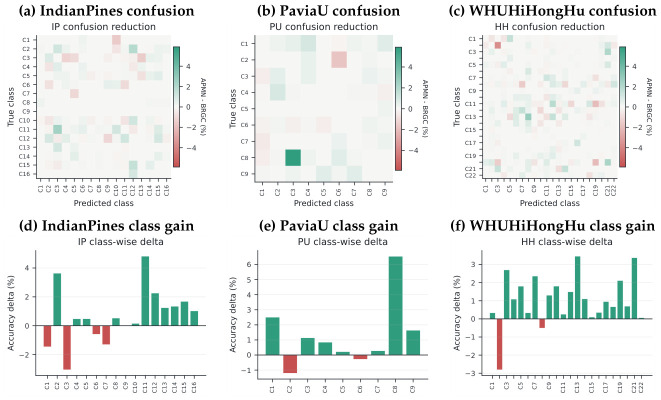
Confusion changes and class-level gains on representative datasets. Panels (**a**–**c**) show the off-diagonal confusion reduction of BRGC relative to APMN on IndianPines, PaviaU, and WHUHiHongHu, respectively. Panels (**d**–**f**) show the corresponding class-wise accuracy delta. Positive green values in the confusion panels indicate that a true-class to wrong-class confusion is reduced by BRGC, whereas negative red/brown values indicate that the confusion is increased. In the class-gain panels, green denotes gains and red denotes losses. This figure identifies the classes and confusion relations that contribute to BRGC gains. Improvements concentrated on fine-grained crops, urban materials, or boundary-complex classes support the mechanism of boundary-risk calibration.

To further examine whether BRGC acts on boundary-confused classes, we aggregate confusion matrices over 10 random seeds on IndianPines, PaviaU, and WHUHiHongHu, and we plot the off-diagonal error change of BRGC relative to APMN. Green values in the first row of Figure 5 indicate inter-class confusions that occur under APMN but are reduced by BRGC. Brown values indicate confusions that are newly introduced or increased by BRGC. The second row gives the class-wise accuracy delta on the same datasets.

Figure 5 shows that BRGC gains are not uniformly distributed across all classes. They concentrate on low-margin, boundary-complex, or inter-class-similar classes. Several crop classes in IndianPines improve substantially, although a few classes decrease. In PaviaU, urban material classes such as Self-Blocking Bricks gain considerably. In WHUHiHongHu, many fine-grained vegetable or crop classes show positive changes, while some individual classes are still damaged. This is consistent with the boundary-risk entanglement defined in Section 3: graph calibration can repair part of the wrong-far hard samples, but it cannot completely avoid damage to risky-far hard samples.

To preserve both class-wise numerical details and cross-dataset trends, we do not merge all class accuracies of the 10 datasets into a single main-text table. Instead, we use a “representative classes in the main text plus complete class-wise tables in the Supplementary Material” design. Table 6 reports representative classes from IndianPines, PaviaU, and WHUHiHongHu that best reflect boundary-risk entanglement. Complete class-wise accuracies for all 10 target datasets are provided in Tables S3–S12. This presentation preserves verifiable class-wise values while preventing the main result table from becoming too wide and dense.

Table 6 further shows that the class-level gains of BRGC are selective. Classes with larger improvements are mostly fine-grained crops, urban materials, or high-resolution crop subtypes, which are directly related to support coverage deficiency and boundary-risk entanglement. At the same time, decreases on Corn mintill, Meadows, and Road indicate that BRGC does not benefit all classes simultaneously. These negative results are retained in the discussion because they define the applicability boundary of graph residual calibration.

### 5.5. Ablation Study and Low-Intervention Diagnosis

The main results and matched-protocol transductive comparisons show that BRGC is effective overall, but they do not isolate which components contribute to the gains or whether the method satisfies the low-intervention calibration objective. To explicitly evaluate the role of each component, we conduct four module-wise ablation experiments under the final BRGC configuration: removing graph residual calibration, removing boundary-aware training, removing low-margin gating, and removing residual clipping. All ablations use the same datasets, 5-shot setting, 10 random seeds, and base feature interface.

Table 7 reports the ablation results. Removing graph residual calibration decreases macro OA from 90.032% to 89.528%, a drop of 0.504 percentage points relative to full BRGC. This indicates that inference-stage target query graph residual calibration is one of the main sources of improvement. Removing boundary-aware training decreases macro OA to 89.779%, a drop of 0.253 percentage points relative to full BRGC. This indicates that training-stage boundary exposure is also beneficial. Both ablations remain above APMN, suggesting that boundary training and graph residual calibration each have positive effects, and that the best result comes from their combination.

The two risk-control ablations require a different interpretation from the two performance-source ablations. Removing the low-margin gate yields the same macro-average accuracy metrics as full BRGC, but it increases the candidate selected ratio from 33.34% to 100.00%. Thus, the gate does not provide an additional accuracy gain under the current fixed configuration; instead, it defines the candidate intervention region and keeps graph residual calibration focused on low-margin samples. Removing residual clipping increases macro OA by only 0.011 percentage points, while the changed and damage ratios also increase slightly. We therefore retain residual clipping not as an accuracy-maximizing component but as a bounded-update risk-control mechanism. Overall, the performance gain mainly comes from boundary exposure training plus graph residual calibration, whereas low-margin gating and residual clipping constrain calibration risk and clarify the method boundary.

The complete ablation diagnostics, including accuracy before and after calibration, are reported in Supplementary Table S1.

**Figure 6:**
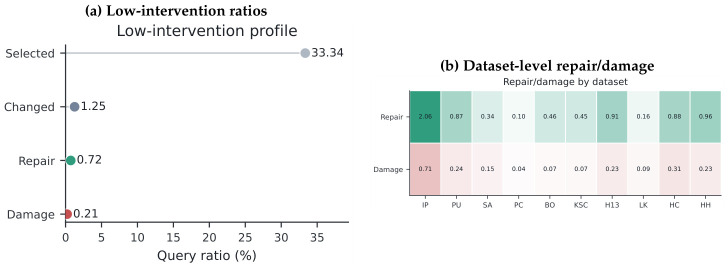
Low-intervention repair/damage diagnosis of BRGC. Panel (**a**) shows selected, changed, repair, and damage ratios. Selected denotes the proportion of queries selected by the low-margin gate, changed denotes the proportion of final labels changed, repair denotes the proportion of samples wrong under APMN but correct under BRGC, and damage denotes the proportion of samples correct under APMN but wrong under BRGC. Panel (**b**) shows repair/damage ratios across datasets. This figure evaluates whether BRGC satisfies the low-intervention calibration objective: changing only a small number of low-margin samples while keeping repair higher than damage.

In addition to component ablations, we further compute selected ratio, changed ratio, repair ratio, and damage ratio. Selected denotes the proportion of queries selected as candidates by the low-margin gate. Changed denotes the proportion of final predicted labels that are changed. Repair denotes the proportion of samples that are wrong under the base classifier but correct under BRGC. Damage denotes the proportion of samples that are correct under the base classifier but wrong under BRGC. Final BRGC selects approximately 33.34% of low-margin queries as candidates but changes only about 1.25% of final query predictions. Its repair ratio is 0.72%, and its damage ratio is 0.21%. This indicates that BRGC does not substantially alter target-domain predictions. It makes conservative corrections on a small number of low-margin samples, and its repair ratio exceeds its damage ratio, yielding a positive net effect. In other words, BRGC adds a limited amount of graph-structure evidence in boundary regions rather than replacing the original support-set decision with graph propagation. Figure 6 summarizes these selected, changed, repair, and damage ratios for diagnosing the low-intervention behavior of BRGC.

### 5.6. Cross-Method Adaptation

BRGC is intended to be a calibration framework at the support-set decision interface, rather than a method-specific trick for one base model. To preliminarily test this interface assumption, we attach the graph residual calibration module to FDFSL and MLPA while preserving their original training procedures and feature extraction pipelines. Table 8 shows that FDFSL-BRGC improves macro OA by +0.222 over FDFSL, and MLPA-BRGC improves macro OA by +0.610 over MLPA.

These results lead to two bounded conclusions. First, BRGC graph residual calibration can be positively adapted to different support-set decision-based methods. MLPA-BRGC outperforms MLPA on all 10 datasets and provides relatively strong cross-method evidence. Second, the gain magnitude differs across base methods, indicating that BRGC still depends on whether the base feature manifold contains repairable low-margin errors. We therefore treat this part as preliminary evidence of framework adaptability rather than extending it into a strong claim of universal effectiveness for all few-shot classifiers.

Cross-base-method visual gains are provided in Supplementary Figure S2. The main text retains only Table 8 because these experiments primarily support interface adaptability, not the central performance claim of the paper.

### 5.7. Parameter Sensitivity and Statistical Significance

Finally, we test whether the improvement of BRGC depends on a single parameter point or a few random seeds. The final main experiments use a unified configuration: k=20, α=0.8, τ=0.05, 30 propagation iterations, λ=0.8, and c=0.1. This configuration comes from a fixed higher-strength calibration setting in the previous parameter sensitivity study and has been rerun as an independent method on all 10 datasets with 10 seeds. The manuscript therefore uses this fixed configuration as BRGC throughout.

**Figure 7:**
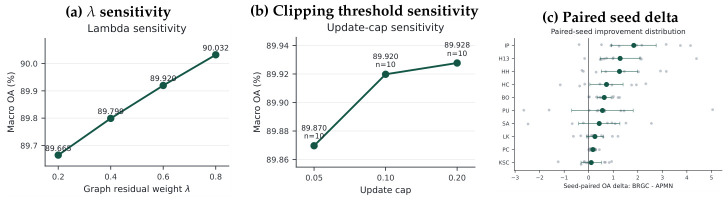
Parameter sensitivity and paired seed significance analysis. Panel (**a**) shows the effect of graph residual weight λ on macro OA and visualizes the trend between calibration strength and performance. Panel (**b**) shows the effect of the residual clipping threshold *c* and checks whether the default clipping is fragile. Panel (**c**) shows paired seed delta and confidence intervals under the same datasets and random seeds. This figure indicates that the improvement of BRGC is not caused by a single random seed or an uninterpretable single parameter point.

To avoid treating the parameter choice as a single favorable setting, we retain sensitivity analysis for λ and *c*. Figure 7 and Table S2 show that as λ increases from 0.2 to 0.8, macro OA monotonically increases from 89.665% to 90.032%, and macro AA and Kappa also improve. For the clipping-threshold subset evaluated at λ=0.6, the macro OA values for c=0.05, c=0.10, and c=0.20 are 89.870%, 89.920%, and 89.928%, respectively, showing only small differences. This result indicates that the graph residual weight controls calibration strength, whereas the residual clipping threshold mainly acts as a risk upper bound.

Although λ=0.8 comes from the sensitivity analysis, we do not report only the highest point in the scan. We implement it as a fixed method and rerun the complete 10-seed experiments. Even under this higher-strength configuration, the changed ratio remains only 1.25%, and the damage ratio is 0.21%, indicating that increased calibration strength does not cause large-scale alteration of predictions. The final BRGC can therefore be interpreted as a fixed configuration with higher calibration strength that still preserves the low-intervention property.

Because cross-domain 5-shot experiments have noticeable seed variance, we further perform paired seed comparisons under the same datasets and random seeds. Table 9 shows that BRGC improves OA over APMN by +0.724 on average, with a 95% confidence interval of [0.480, 0.968]. Both the paired t-test and Wilcoxon test are significant. BRGC also significantly outperforms iLPC and TIM. Compared with Graph LP and LaplacianShot, BRGC has positive average paired OA differences, although the confidence intervals cross zero. The statistical results therefore support the main claim that BRGC improves its base classifier and provides competitive risk-controlled calibration while also delimiting the cases in which full graph propagation remains comparable. Compared with the weaker λ=0.6 calibration configuration, final BRGC still achieves a paired gain of +0.112 and higher OA in 95 of 100 paired runs.

### 5.8. Computational Complexity and Time Cost

We analyze the additional computational cost introduced by BRGC at the support-set decision interface. Let *n* denote the number of target support and query samples in an inference episode, *d* the feature dimension, $C_{t}$ the number of target classes, *k* the graph degree, and *T* the number of graph-propagation iterations. After feature extraction, the base support-set classifier computes class scores with a cost proportional to the support-query scoring operation. BRGC adds three inference-time operations: direct k-nearest-neighbor graph construction with complexity $O(n^{2}d)$, support-clamped sparse graph propagation with complexity $O(TnkC_{t})$, and residual gating, normalization, and clipping with complexity $O(nC_{t})$. The additional memory cost is $O(nk+nC_{t})$ for sparse graph edges and score matrices. Because BRGC does not redesign the backbone network, its main overhead is concentrated in inference-time graph residual calibration rather than in a heavier feature extractor.

Table 10 reports the measured time cost from the original 10-dataset × 10-seed main-experiment logs. BRGC and APMN have the same order of training time under the same 5000-episode setting, whereas BRGC adds an average graph-calibration cost of 30.953 s during testing. The small measured difference in training time is not interpreted as a speed advantage because the runs were scheduled independently; the main additional cost of BRGC is the inference-time graph calibration step. The average total test time increases from 50.646 s for APMN to 68.510 s for BRGC. Across the 100 BRGC runs, graph-calibration time ranges from 0.388 s to 135.062 s because target image sizes and graph sizes differ across datasets.

### 5.9. Summary of Experimental Findings

The experiments support three conclusions. First, under the target-unlabeled transductive protocol, BRGC consistently improves its base support-set classifier APMN and achieves the highest macro-average OA, AA, and Kappa among matched-protocol transductive baselines. Second, classification maps, confusion changes, and class-level results show that the main gains of BRGC come from local repair in boundary-complex and inter-class-similar classes, not from uniform improvements across all classes. Third, component ablations and repair/damage diagnostics show that boundary-aware training and graph residual calibration are the main performance sources, while low-margin gating and residual clipping mainly define the low-intervention region and risk upper bound. Adaptation results on FDFSL and MLPA suggest interface-level transferability, but this conclusion should remain limited to support-set decision-based classifiers and the current validation scope.

## 6. Discussion

The results of BRGC suggest that the residual errors of cross-domain few-shot HSI cannot be fully attributed to insufficient backbone representation. For support-set decision-based classifiers, the target-domain 5-shot support samples provide only sparse class evidence. When a class contains multiple spectral–spatial sub-modes, the low-margin boundary region can contain both repairable errors and high-risk correct samples. BRGC converts this phenomenon into a decision-level calibration problem. It exposes class conflicts through boundary mixing during training, uses target unlabeled graph structure as complementary evidence during inference, and finally corrects original scores only through low-margin, bounded residual updates.

This positioning also explains the relation between BRGC and standard transductive graph methods. The results of Graph LP and LaplacianShot show that the target query graph itself is already an informative transductive signal. BRGC differs by preserving the original base-classifier scores and constraining graph propagation output as a risk-controlled residual signal. The ablation results in Table 7 are consistent with this interpretation. Boundary-aware training and graph residual calibration contribute the main performance gains, whereas low-margin gating and residual clipping delimit candidate regions and update magnitude. In other words, the core value of BRGC is to convert the query graph from an alternative decision source into low-intervention calibration evidence.

Parameter sensitivity and cross-method adaptation further define the boundary of the framework. Final BRGC uses λ=0.8 and has been rerun on all datasets with 10 seeds, so the main results are not a single point selected from a sensitivity table. At the same time, the changed ratio remains at 1.25%, indicating that higher calibration strength does not cause large-scale alteration of predictions. Positive adaptation results on FDFSL and MLPA indicate that BRGC can be attached to different support-set decision interfaces, but the gain magnitude differs. This suggests that BRGC depends on whether the base feature manifold contains repairable low-margin errors.

BRGC also has clear limitations. First, it is a target-unlabeled transductive method. Inference requires access to the unlabeled features of the entire target query set, so it should not be mixed with strict inductive few-shot methods without protocol annotation. Second, the graph structure is still based on base feature distance. When feature distance is severely inconsistent with semantic distance, graph calibration may fail. Third, wrong-far hard samples and risky-far hard samples remain highly entangled from an unlabeled perspective. The current low-margin gate can reduce intervention risk but cannot fully identify the correction direction. Fourth, full graph propagation remains competitive on some target domains, especially when the feature graph is already reliable. BRGC is therefore best interpreted as a conservative reliability-calibration strategy that trades aggressive propagation for bounded residual correction. Future work can explore class-pair adaptive gating, uncertainty estimation, multi-view spectral-spatial graphs, and more reliable counterfactual risk discrimination.

## 7. Conclusions

This paper proposed BRGC, a low-intervention boundary-risk graph calibration framework for cross-domain few-shot HSI classification. Starting from boundary-risk entanglement in support-set decisions, BRGC combines boundary-aware mixability training with risk-gated graph residual calibration at inference time. It preserves the original scores of the base classifier while using the target unlabeled query graph for small corrections.

On 10 target datasets and 10 random seeds, BRGC consistently improves its base support classifier and achieves competitive macro-average results among matched-protocol transductive baselines. Component ablations, repair/damage diagnostics, parameter sensitivity, and paired seed tests show that the main gain comes from the combination of boundary exposure training and graph residual calibration, while low-margin gating and residual clipping define the calibration range and risk upper bound. Adaptation results on FDFSL and MLPA further suggest that BRGC has transfer potential at the support-set decision interface, although its gains remain limited by the quality of the base feature manifold and the number of repairable low-margin errors. Overall, BRGC provides a decision-reliability route for scarce-label hyperspectral image interpretation by improving support-set classifiers through risk-controlled calibration.

## Figures and Tables

**Table 1:** Mechanism-level differences between Boundary-Risk Graph Calibration (BRGC) and related methods. APMN denotes attention-based patch mixing network, TIM denotes transductive information maximization, and protoLP denotes prototype-based label propagation.

Method	Boundary Train	Query Graph	Base Score Kept	Bounded Residual	Low-Margin Gate	Support Clamp	Role
APMN	Yes	No	Yes	No	No	Yes	Boundary representation
Standard Graph LP	No	Yes	No	No	No	Usually	Direct propagation
TIM/prototype rectification	No	Yes	No	No	No	Partly	Transductive adaptation
protoLP	No	Yes	No	No	No	Yes	Prototype-LP refinement
BRGC	Yes	Yes	Yes	Yes	Yes	Yes	Low-intervention calibration

**Table 2:** Experimental protocol summary.

Method Type	Representative Methods	Source	Support	Unlabeled Query	Query Labels	Protocol Role
Target-only refs.	SVM, 3DCNN, SSRN	No	Yes	No	No	protocol reference
Inductive CD-FSL	DCFSL, Gia-CFSL, FDFSL, MLPA, APMN	Yes	Yes	No	No	support-only inference
Matched transductive	Graph LP, LaplacianShot, TIM, iLPC	Yes	Yes	Yes	No	same query access
BRGC protocol	BRGC	Yes	Yes	Yes	No	residual calibration

**Table 3:** Decision mechanisms and key assumptions of matched-protocol transductive methods.

Method	Base Input	Query Use	Update Form	Main Risk
Graph LP	APMN feat. + support labels	support-query kNN	direct propagated score	erroneous-edge spread
LaplacianShot	APMN unary + query kNN	query Laplacian	graph-smoothed labels	over-smoothing
TIM	APMN score + query pred.	entropy/marginal prior	transductive re-optimization	class-prior mismatch
iLPC	APMN score + query graph	pseudo-label propagation	iterative refinement	error reinforcement
BRGC	APMN score + support-query graph	support-clamped graph	gated clipped residual	low repair recall

**Table 4:** OA comparison on 10 target datasets. Values are mean ± std. The best and second-best results in each row are bolded and underlined, respectively.

Dataset	DCFSL	Gia-CFSL	FDFSL	MLPA	APMN	BRGC
Botswana	96.61 ± 1.25	96.54 ± 1.34	97.26 ± 1.36	96.88 ± 1.17	97.89 ± 1.10	**98.53 ± 0.97**
Houston13	75.81 ± 2.00	76.13 ± 1.66	78.26 ± 2.74	77.76 ± 2.14	81.05 ± 2.07	**82.33 ± 2.15**
IndianPines	63.71 ± 3.06	65.99 ± 3.91	69.85 ± 2.56	68.56 ± 2.88	76.66 ± 1.81	**78.49 ± 1.94**
KSC	88.54 ± 1.98	90.86 ± 1.43	93.56 ± 1.79	92.39 ± 1.34	96.42 ± 1.24	**96.52 ± 1.67**
PaviaC	97.20 ± 0.53	96.91 ± 0.69	97.55 ± 0.37	96.97 ± 0.57	97.99 ± 0.36	**98.16 ± 0.40**
PaviaU	80.64 ± 2.96	79.60 ± 3.06	82.54 ± 3.38	81.26 ± 2.81	88.78 ± 3.25	**89.34 ± 3.01**
Salinas	88.93 ± 1.43	89.45 ± 1.66	91.23 ± 1.19	90.15 ± 1.19	91.93 ± 1.33	**92.36 ± 1.49**
WHUHiHanChuan	68.31 ± 2.79	67.70 ± 4.08	68.57 ± 2.88	73.53 ± 2.52	80.45 ± 1.94	**81.17 ± 2.08**
WHUHiHongHu	74.03 ± 2.89	76.23 ± 1.96	76.34 ± 2.19	78.77 ± 2.40	86.09 ± 1.06	**87.34 ± 1.25**
WHUHiLongKou	94.10 ± 1.83	93.07 ± 1.48	94.77 ± 1.86	94.45 ± 1.77	95.83 ± 1.15	**96.08 ± 1.19**

**Table 5:** Macro-average OA, AA, and Kappa comparison among methods and matched-protocol transductive baselines. For performance columns where larger values indicate better results, the best and second-best results are bolded and underlined, respectively. Tied second-best results are both underlined.

Method	Protocol/Role	Macro OA	Macro AA	Macro Kappa	Macro OA vs. APMN
DCFSL	strict inductive	82.788	82.062	79.946	−6.520
Gia-CFSL	strict inductive	83.246	82.768	80.432	−6.061
FDFSL	strict inductive	84.993	84.776	82.493	−4.315
MLPA	strict inductive	85.071	84.700	82.561	−4.237
APMN	inductive support decision	89.308	89.548	87.517	+0.000
Graph LP	transductive LP	89.887	90.115	88.189	+0.580
LaplacianShot	transductive Laplacian	89.888	90.112	88.189	+0.580
TIM	transductive entropy	81.548	87.336	78.738	−7.760
iLPC	transductive iLPC	89.568	89.814	87.818	+0.260
BRGC	risk-gated residual	**90.032**	**90.278**	**88.353**	**+0.724**

**Table 6:** Class-wise accuracy changes of representative boundary classes.

Dataset	Class	APMN	BRGC	Δ	pos./neg./tie Seeds
IndianPines	Soybean mintill	70.66	75.47	+4.82 ± 5.34	9/1/0
IndianPines	Corn notill	64.13	67.76	+3.63 ± 6.50	7/3/0
IndianPines	Corn mintill	65.88	62.82	−3.05 ± 9.90	4/6/0
PaviaU	Self-Blocking Bricks	80.20	86.74	+6.54 ± 9.75	8/2/0
PaviaU	Asphalt	86.16	88.67	+2.51 ± 4.21	7/3/0
PaviaU	Meadows	89.83	88.62	−1.21 ± 4.63	4/6/0
WHUHiHongHu	Small Brassica chinensis	58.99	62.45	+3.46 ± 6.36	8/2/0
WHUHiHongHu	Broad bean	89.98	93.36	+3.38 ± 7.67	6/4/0
WHUHiHongHu	Road	81.38	78.57	−2.81 ± 8.87	5/5/0

**Table 7:** Module-wise ablation study and low-intervention diagnosis of BRGC. The four ablation variants remove graph residual calibration, boundary-aware training, low-margin gating, and residual clipping, respectively, while keeping the same datasets, 5-shot protocol, 10 random seeds, and base feature interface. Accuracy and gain columns evaluate performance contribution, whereas Selected, Changed, Repair, and Damage diagnose intervention behavior. For accuracy and gain columns where larger values indicate better results, the best and second-best results are bolded and underlined, respectively; tied second-best results are both underlined. Diagnostic ratios are not highlighted because they reflect intervention behavior rather than a single monotonic performance objective.

Config.	Role	OA	AA	Kappa	vs. APMN	vs. BRGC	Selected	Changed	Repair	Damage
APMN	base	89.308	89.548	87.517	+0.000	−0.724	–	–	–	–
BRGC	full	90.032	90.278	88.353	+0.724	+0.000	33.34%	1.25%	0.72%	0.21%
no graph calib.	boundary only	89.528	89.785	87.772	+0.220	−0.504	0.00%	0.00%	0.00%	0.00%
no boundary train	graph only	89.779	90.017	88.063	+0.471	−0.253	33.34%	1.28%	0.71%	0.24%
no margin gate	ungated	90.032	90.278	88.353	+0.724	+0.000	100.00%	1.25%	0.72%	0.21%
no clipping	unclipped	**90.043**	**90.284**	**88.365**	**+0.735**	**+0.011**	33.34%	1.27%	0.73%	0.22%

**Table 8:** Transfer results of BRGC graph residual calibration on different base methods. The best and second-best results in each numerical column are bolded and underlined, respectively.

Base Method	Base Macro OA	BRGC-Adapted Macro OA	Gain	Dataset Win Rate
FDFSL	84.993	85.215	+0.222	8/10
MLPA	**85.071**	**85.681**	**+0.610**	**10/10**

**Table 9:** Paired seed significance tests. For directionally comparable columns, namely mean OA difference and wins, the best and second-best results are bolded and underlined, respectively. Significance-test columns are not highlighted because smaller *p*-values are used for hypothesis testing rather than ranking method performance.

Comparison	Mean OA Difference	95% CI	Paired *t*-Test	Wilcoxon	Wins
BRGC vs. APMN	+0.724	[+0.480, +0.968]	$p=5.24\times 10^{-8}$	$p=2.45\times 10^{-9}$	77/100
BRGC vs. Graph LP	+0.145	[−0.087, +0.377]	$p=2.18\times 10^{-1}$	$p=2.69\times 10^{-1}$	55/100
BRGC vs. LaplacianShot	+0.144	[−0.088, +0.376]	$p=2.21\times 10^{-1}$	$p=2.76\times 10^{-1}$	55/100
BRGC vs. TIM	**+8.484**	[+7.128, +9.840]	$p=6.82\times 10^{-22}$	$p=3.90\times 10^{-18}$	**100/100**
BRGC vs. iLPC	+0.464	[+0.229, +0.699]	$p=1.64\times 10^{-4}$	$p=2.13\times 10^{-5}$	68/100
BRGC vs. BRGC (λ=0.6)	+0.112	[+0.091, +0.134]	$p=3.65\times 10^{-17}$	$p=4.20\times 10^{-17}$	95/100

**Table 10:** Computational complexity and measured time cost of BRGC.

Stage	Main Operation	Complexity	Measured Time
Training	5000-episode episodic training with the same backbone interface	Same backbone training order; boundary-aware mixing adds data construction and loss terms	APMN: 1237.354 s; BRGC: 1172.381 s
Base inference	Support-set class-score computation after feature extraction	Proportional to base support-query scoring, e.g., $O(nC_{t}d)$ after feature extraction	APMN total test: 50.646 s
Graph calibration	k-nearest-neighbor graph construction and support-clamped propagation	$O\left(n^{2}d\right)+O\left(TnkC_{t}\right)+O\left(nC_{t}\right)$	BRGC calibration: 30.953 s
BRGC total test	Base inference plus risk-gated graph residual calibration	Base inference plus BRGC calibration overhead	BRGC total test: 68.510 s

## Data Availability

The hyperspectral datasets used in this study are publicly available from their original data providers, including the EHU/GIC Hyperspectral Remote Sensing Scenes, the University of Houston IEEE GRSS Data Fusion Contest data resources, and the WHU-Hi dataset resource page, as cited in the manuscript. To respect the original data distribution policies, the raw hyperspectral datasets are not redistributed with this manuscript. The source code, configuration files, evaluation scripts, and processed result summaries will be made available in a public repository after acceptance. Before public release, these materials are available from the corresponding author upon reasonable request for review and reproducibility checks. Additional training logs and large intermediate files are available from the corresponding author upon reasonable request.

## Supplementary Materials

The following supporting information can be downloaded at: https://www.mdpi.com/article/10.3390/s26154903/s1. Figure S1: Local zoom-in classification results on representative target datasets; Figure S2: Adaptation of BRGC to different base methods; Table S1: Complete BRGC module-wise ablation study and calibration diagnostics; Table S2: BRGC parameter sensitivity results; Tables S3–S12: complete class-wise accuracies for Botswana, Houston13, IndianPines, KSC, PaviaC, PaviaU, Salinas, WHUHiHanChuan, WHUHiHongHu, and WHUHiLongKou; Table S13: Dataset-level OA of matched-protocol transductive baselines.

## Abbreviations

The following abbreviations are used in this manuscript:

HSI	Hyperspectral image
FSL	Few-shot learning
BRGC	Boundary-risk graph calibration
APMN	Attention-based patch mixing network
OA	Overall accuracy
AA	Average accuracy
