# Supplementary material for: Low-Intervention Boundary-Risk Graph Calibration for Cross-Domain Few-Shot Hyperspectral Image Classification
Source: Sensors (Basel). 2026 Aug 3;26(15):4903. doi: 10.3390/s26154903 (PMC13470040; doi:10.3390/s26154903)
Supplement: Supplementary file 1 [file sensors-26-04903-s001.zip › sensors-4453171-supplementary.pdf]

Supplementary Material: Low-Intervention Boundary-Risk Graph Calibration for Cross-Domain Few-Shot Hyperspectral Image Classification

This supplementary material corresponds to the main manuscript "Low-Intervention Boundary-Risk Graph Calibration for Cross-Domain Few-Shot Hyperspectral Image Classification". It contains local zoom-in maps, complete ablation diagnostics, parameter sensitivity, full class-wise accuracies, and dataset-level results for matched-protocol transductive baselines. References are provided in the main manuscript.

Supplementary Experimental Materials

A. Local Zoom-In Maps

Supplementary Figure S1 shows local zoom-in results on representative datasets. For each dataset, the zoom window is automatically selected from the error-change map by prioritizing regions with dense repaired pixels, namely pixels that are wrong under APMN but correct under BRGC. This figure complements Figure 4 in the main text and further examines whether BRGC repairs occur near field edges, urban material interfaces, and fine-grained crop patches.

Dataset	GT locator	APMN local map	BRGC local map	Local error change

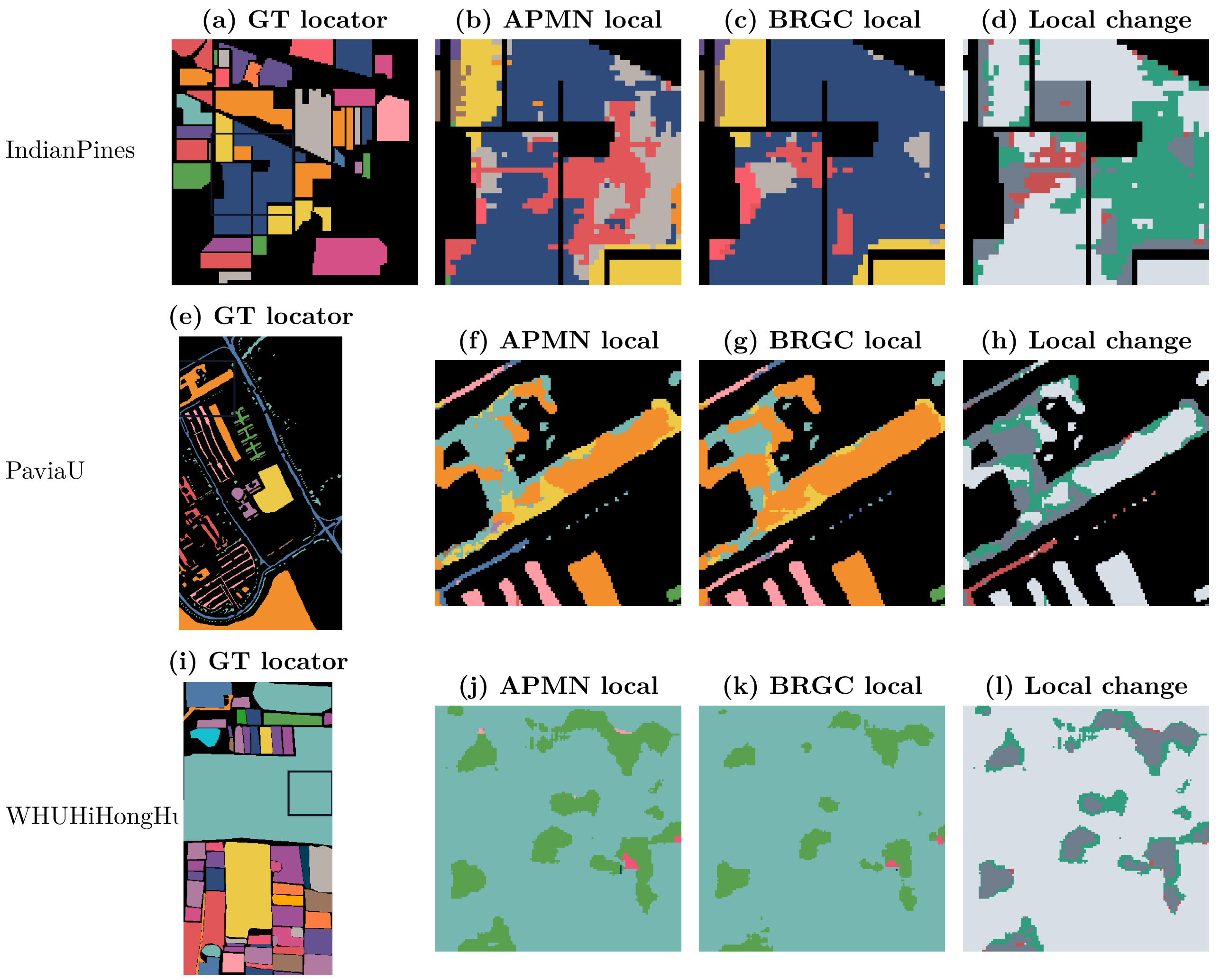

Supplementary Figure S1. Local zoom-in classification results on representative target datasets. Panels (a)–(d), (e)–(h), and (i)–(l) correspond to IndianPines, PaviaU, and WHUHiHongHu, respectively. Within each dataset, the four panels show the zoom-window location in the ground-truth map, the local APMN

classification map, the local BRGC classification map, and the local error-change map. The first three columns are used to compare local class predictions with the ground truth. The last column represents correctness changes rather than class labels: black denotes unlabeled or non-evaluated background, light gray denotes pixels correctly predicted by both methods, dark gray denotes pixels incorrectly predicted by both methods, green denotes pixels repaired by BRGC, and red denotes newly introduced errors. This supplementary figure magnifies local boundary repair phenomena that are difficult to see in Fig. 4 of the main text.

B. Complete Ablation Study and Low-Intervention Diagnostics

Table S1 provides the complete ablation study corresponding to Table 7 in the main text. It evaluates the full BRGC model and four module-removal variants: without graph residual calibration, without boundary-aware training, without low-margin gating, and without residual clipping. Table 7 in the main text retains the macro-average metrics and key repair/damage conclusions, while Table S1 additionally reports the selected ratio to distinguish candidate samples from final prediction changes. After removing the low-margin gate, the selected ratio increases from 33.34% to 100.00%, but the final changed, repair, and damage ratios remain almost unchanged. This indicates that, under the current fixed configuration, final label changes are mainly determined by the direction and magnitude of graph residuals. The primary role of the low-margin gate is to define a low-intervention candidate region, rather than to provide an additional accuracy gain. Removing residual clipping slightly increases macro OA by 0.011 percentage points, but also slightly increases changed and damage ratios. We therefore retain the cap as a risk upper bound rather than using the variant without residual clipping as the main method.

Table S1. Complete BRGC module-wise ablation study and calibration diagnostics

Configuration	Macro OA	Macro AA	Macro Kappa	Accuracy before calibration	Accuracy after calibration	Calibratio gain	Selected	Changed	Repair	Damage
BRGC	90.032	90.278	88.353	89.528	90.032	+0.504	33.34%	1.25%	0.72%	0.21%
w/o graph residual calibration	89.528	89.785	87.772	89.528	89.528	+0.000	0.00%	0.00%	0.00%	0.00%
w/o boundary-aware training	89.779	90.017	88.063	89.308	89.779	+0.471	33.34%	1.28%	0.71%	0.24%
w/o low-margin gate	90.032	90.278	88.353	89.528	90.032	+0.504	100.00%	1.25%	0.72%	0.21%
w/o residual clipping	90.043	90.284	88.365	89.528	90.043	+0.515	33.34%	1.27%	0.73%	0.22%

C. Cross-Method Adaptation Visualization

Supplementary Figure S2 shows the macro OA differences and dataset-level OA gains of FDFSL, MLPA, and their BRGC-adapted versions. This figure complements Table 8 in the main text and shows that BRGC can be attached to different support-set decision interfaces, although the gain magnitude varies across base methods.

Cross-base-method macro OA	Dataset-level gain
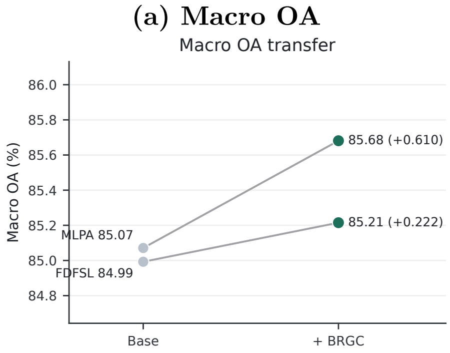	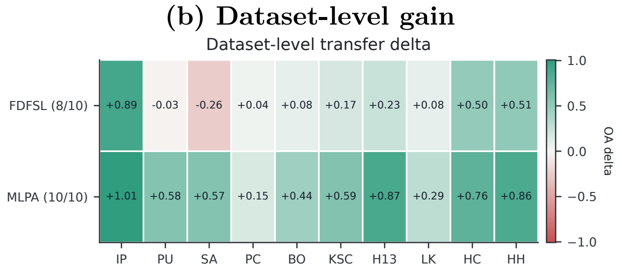

Supplementary Figure S2. Adaptation of BRGC to different base methods. Panel (a) shows macro OA differences between FDFSL, MLPA, and their BRGC-adapted versions, where upward lines indicate performance improvement after BRGC adaptation. Panel (b) shows OA gains on each target dataset, with green denoting positive gains and red denoting negative gains. This figure complements Table 8 in the main text and indicates that BRGC can be attached to different support-set decision interfaces, while its gains depend on the base feature space and the repairable-error structure of the target dataset.

D. Complete Parameter Sensitivity Table

Table S2 reports the complete sensitivity results for the graph residual weight λ and residual clipping threshold c. Fig. 7 in the main text shows the trend, while Table S2 preserves the exact values. Final BRGC uses $\lambda = 0.8, c = 0.10$, so "OA relative to final BRGC" uses this configuration as the zero point.

Table S2. BRGC parameter sensitivity results

Configuration	Macro OA	Macro AA	Macro Kappa	OA relative to final BRGC	Changed	Repair	Damage
$\lambda = 0.2, c = 0.10$	89.665	89.924	87.929	-0.367	0.32%	0.19%	0.05%
$\lambda = 0.4, c = 0.10$	89.799	90.061	88.084	-0.233	0.63%	0.37%	0.10%
$\lambda = 0.6, c = 0.10$	89.920	90.178	88.224	-0.112	0.94%	0.55%	0.16%
$\lambda = 0.8, c = 0.10$	**90.032**	**90.278**	**88.353**	**+0.000**	1.25%	0.72%	0.21%
$\lambda = 0.6, c = 0.05$	89.870	90.128	88.167	-0.162	0.85%	0.48%	0.14%
$\lambda = 0.6, c = 0.20$	89.928	90.181	88.232	-0.104	0.96%	0.56%	0.16%

E. Complete Class-Wise Accuracy Tables

Tables S3-S12 report complete class-wise accuracies for all target datasets. Each table lists APMN accuracy, BRGC accuracy, class-level change Δ, standard deviation of Δ across seeds, and the numbers of positive, negative, and tied seeds. Table 6 in the main text includes only representative boundary classes for the main narrative. The complete class-wise tables ensure that all class results are traceable and that the paper does not report only classes that benefit.

Supplementary table	Dataset	Content
S3	Botswana	Class-wise APMN/BRGC accuracies and changes for all 14 classes
S4	Houston13	Class-wise APMN/BRGC accuracies and changes for all 15 classes
S5	IndianPines	Class-wise APMN/BRGC accuracies and changes for all 16 classes
S6	KSC	Class-wise APMN/BRGC accuracies and changes for all 13 classes
S7	PaviaC	Class-wise APMN/BRGC accuracies and changes for all 9 classes
S8	PaviaU	Class-wise APMN/BRGC accuracies and changes for all 9 classes
S9	Salinas	Class-wise APMN/BRGC accuracies and changes for all 16 classes
S10	WHUHiHanChuan	Class-wise APMN/BRGC accuracies and changes for all 16 classes
S11	WHUHiHongHu	Class-wise APMN/BRGC accuracies and changes for all 22 classes
S12	WHUHiLongKou	Class-wise APMN/BRGC accuracies and changes for all 9 classes

Table S3. Botswana Class-Wise Accuracy

Class ID	Class	APMN	BRGC	Δ	+/-/0 seeds
1	Water	97.28	98.72	+1.43 ± 2.12	7/2/1
2	Hippo grass	100.00	100.00	+0.00 ± 0.00	0/0/10
3	Floodplain grasses 1	98.37	99.15	+0.77 ± 1.63	2/0/8
4	Floodplain grasses 2	99.00	99.67	+0.67 ± 1.67	3/1/6
5	Reeds	95.15	96.40	+1.25 ± 4.11	5/3/2
6	Riparian	94.13	96.06	+1.93 ± 4.38	8/2/0
7	Firescar	99.41	100.00	+0.59 ± 1.25	3/0/7
8	Island interior	98.89	99.44	+0.56 ± 2.76	1/1/8
9	Acacia woodlands	98.80	98.96	+0.16 ± 3.85	3/1/6
10	Acacia shrublands	100.00	100.00	+0.00 ± 0.00	0/0/10
11	Acacia grasslands	95.47	96.23	+0.77 ± 3.28	4/2/4
12	Short mopane	100.00	99.77	-0.23 ± 0.72	0/1/9
13	Mixed mopane	99.81	100.00	+0.19 ± 0.60	1/0/9
14	Exposed soils	95.78	94.78	-1.00 ± 5.77	1/2/7

Table S4. Houston13 Class-Wise Accuracy

Class ID	Class	APMN	BRGC	Δ	+/-/0 seeds

1	Healthy grass	85.40	85.16	-0.24 ± 3.46	6/3/1
2	Stressed grass	90.03	92.25	+2.22 ± 4.05	8/2/0
3	Synthetic grass	98.60	99.13	+0.53 ± 1.92	5/3/2
4	Trees	86.37	91.19	+4.83 ± 10.42	9/1/0
5	Soil	99.53	99.47	-0.06 ± 0.56	5/4/1
6	Water	86.75	88.94	+2.19 ± 4.11	6/1/3
7	Residential	79.40	82.40	+3.00 ± 4.23	7/2/1
8	Commercial	56.67	54.58	-2.09 ± 6.29	3/6/1
9	Road	75.24	75.31	+0.06 ± 4.39	6/4/0
10	Highway	69.75	70.13	+0.38 ± 5.53	6/4/0
11	Railway	72.98	76.99	+4.02 ± 7.98	7/3/0
12	Parking lot 1	65.88	65.97	+0.09 ± 5.48	6/4/0
13	Parking lot 2	92.93	93.75	+0.82 ± 1.90	6/3/1
14	Tennis court	99.88	99.95	+0.07 ± 0.42	1/1/8
15	Running track	93.73	97.53	+3.80 ± 7.57	7/0/3

Table S5. IndianPines Class-Wise Accuracy

Class ID	Class	APMN	BRGC	Δ	+/-/0 seeds
1	Alfalfa	99.51	98.05	-1.46 ± 5.54	1/1/8
2	Corn notill	64.13	67.76	+3.63 ± 6.50	7/3/0
3	Corn mintill	65.88	62.82	-3.05 ± 9.90	4/6/0
4	Corn	95.00	95.47	+0.47 ± 3.83	4/4/2
5	Grass pasture	77.64	78.12	+0.48 ± 4.48	4/6/0
6	Grass trees	92.32	91.72	-0.59 ± 4.99	6/4/0
7	Grass pasture mowed	100.00	98.70	-1.30 ± 4.12	0/1/9
8	Hay windrowed	99.03	99.56	+0.53 ± 0.80	6/0/4
9	Oats	99.33	99.33	+0.00 ± 0.00	0/0/10
10	Soybean notill	71.83	71.99	+0.16 ± 3.27	6/4/0
11	Soybean mintill	70.66	75.47	+4.82 ± 5.34	9/1/0
12	Soybean clean	61.65	63.91	+2.26 ± 8.51	6/4/0
13	Wheat	97.15	98.40	+1.25 ± 2.73	4/1/5
14	Woods	88.36	89.70	+1.34 ± 3.45	6/4/0
15	Buildings Grass Trees Drives	92.57	94.25	+1.68 ± 7.90	7/3/0
16	Stone Steel Towers	93.98	95.00	+1.02 ± 3.41	5/1/4

Table S6. KSC Class-Wise Accuracy

Class ID	Class	APMN	BRGC	Δ	+/-/0 seeds
1	Scrub	98.13	98.73	+0.60 ± 2.40	5/1/4
2	Willow swamp	92.48	91.34	-1.13 ± 2.71	3/4/3
3	Cabbage palm hammock	98.05	99.52	+1.47 ± 1.79	6/0/4

4	Cabbage palm oak hammock	69.60	71.58	+1.98 ± 5.53	7/3/0
5	Slash pine	92.31	90.51	-1.79 ± 9.90	4/4/2
6	Oak broadleaf hammock	99.20	99.02	-0.18 ± 0.74	1/1/8
7	Hardwood swamp	100.00	100.00	+0.00 ± 0.00	0/0/10
8	Graminoid marsh	97.42	97.35	-0.07 ± 2.86	6/1/3
9	Spartina marsh	93.40	92.41	-0.99 ± 3.89	2/3/5
10	Cattail marsh	99.95	100.00	+0.05 ± 0.11	2/0/8
11	Salt marsh	98.65	99.32	+0.68 ± 1.53	4/1/5
12	Mud flats	98.45	98.55	+0.10 ± 1.28	4/3/3
13	Water	100.00	100.00	+0.00 ± 0.00	0/0/10

Table S7. PaviaC Class-Wise Accuracy

Class ID	Class	APMN	BRGC	Δ	+/-/0 seeds
1	Water	99.89	99.95	+0.06 ± 0.14	4/1/5
2	Trees	94.19	94.85	+0.66 ± 1.24	7/3/0
3	Asphalt	90.86	90.76	-0.10 ± 2.58	4/6/0
4	Self-Blocking Bricks	94.61	96.17	+1.56 ± 3.19	5/4/1
5	Bitumen	91.53	91.33	-0.20 ± 1.86	5/5/0
6	Tiles	97.14	97.71	+0.58 ± 0.67	8/2/0
7	Shadows	88.46	88.76	+0.30 ± 2.00	6/4/0
8	Meadows	99.43	99.59	+0.16 ± 0.38	7/3/0
9	Bare Soil	95.31	95.28	-0.03 ± 2.09	5/5/0

Table S8. PaviaU Class-Wise Accuracy

Class ID	Class	APMN	BRGC	Δ	+/-/0 seeds
1	Asphalt	86.16	88.67	+2.51 ± 4.21	7/3/0
2	Meadows	89.83	88.62	-1.21 ± 4.63	4/6/0
3	Gravel	80.12	81.26	+1.14 ± 5.64	4/6/0
4	Trees	92.54	93.39	+0.85 ± 2.89	7/3/0
5	Painted metal sheets	99.65	99.87	+0.22 ± 0.49	4/1/5
6	Bare Soil	90.50	90.21	-0.28 ± 5.87	6/4/0
7	Bitumen	98.62	98.90	+0.28 ± 1.01	5/3/2
8	Self-Blocking Bricks	80.20	86.74	+6.54 ± 9.75	8/2/0
9	Shadows	88.64	90.28	+1.63 ± 2.85	7/2/1

Table S9. Salinas Class-Wise Accuracy

Class ID	Class	APMN	BRGC	Δ	+/-/0 seeds
1	Brocoli green weeds 1	99.27	99.40	+0.13 ± 0.96	4/2/4
2	Brocoli green weeds 2	99.30	99.99	+0.69 ± 2.17	2/0/8
3	Fallow	96.09	93.99	-2.10 ± 6.20	3/2/5
4	Fallow rough plow	99.43	99.96	+0.53 ± 1.07	4/1/5
5	Fallow smooth	90.30	92.14	+1.84 ± 2.85	7/3/0
6	Stubble	98.19	98.84	+0.64 ± 1.29	6/1/3
7	Celery	99.05	99.68	+0.63 ± 1.33	5/0/5
8	Grapes untrained	79.05	79.54	+0.49 ± 7.61	6/4/0
9	Soil vinyard develop	99.92	99.97	+0.05 ± 0.09	6/1/3
10	Corn senesced green weeds	92.70	90.71	-1.99 ± 2.93	3/7/0
11	Lettuce romaine 4wk	99.04	99.29	+0.24 ± 0.80	4/2/4
12	Lettuce romaine 5wk	97.47	97.54	+0.07 ± 1.70	4/5/1
13	Lettuce romaine 6wk	98.41	99.00	+0.59 ± 1.15	7/1/2
14	Lettuce romaine 7wk	96.91	97.77	+0.86 ± 1.96	6/2/2
15	Vinyard untrained	84.42	86.14	+1.73 ± 4.29	6/4/0
16	Vinyard vertical trellis	98.40	98.56	+0.16 ± 1.31	5/4/1

Table S10. WHUHiHanChuan Class-Wise Accuracy

Class ID	Class	APMN	BRGC	Δ	+/-/0 seeds
1	Strawberry	85.11	86.20	+1.09 ± 5.54	5/5/0
2	Cowpea	61.90	62.13	+0.23 ± 4.95	4/6/0
3	Soybean	86.31	89.26	+2.94 ± 3.13	9/1/0
4	Sorghum	98.60	98.58	-0.02 ± 1.20	6/4/0
5	Water spinach	96.74	96.89	+0.14 ± 3.07	4/6/0
6	Watermelon	59.59	62.94	+3.35 ± 8.53	5/5/0
7	Greens	88.50	88.18	-0.32 ± 6.38	5/5/0
8	Trees	58.68	60.30	+1.62 ± 9.16	6/4/0
9	Grass	58.63	54.24	-4.38 ± 8.17	3/7/0
10	Red roof	87.16	89.97	+2.80 ± 7.03	5/5/0
11	Gray roof	81.72	84.34	+2.62 ± 8.79	6/4/0
12	Plastic	81.49	78.75	-2.74 ± 6.60	5/5/0
13	Bare soil	44.25	45.67	+1.42 ± 14.70	5/5/0
14	Road	71.97	74.38	+2.42 ± 12.03	5/5/0

15	Bright object	85.99	88.15	+2.16 ± 5.80	7/3/0
16	Water	94.60	94.29	-0.30 ± 1.87	4/6/0

Table S11. WHUHiHongHu Class-Wise Accuracy

Class ID	Class	APMN	BRGC	Δ	+/-/0 seeds
1	Red roof	87.78	88.11	+0.33 ± 4.28	6/4/0
2	Road	81.38	78.57	-2.81 ± 8.87	5/5/0
3	Bare soil	82.63	85.33	+2.70 ± 6.22	8/2/0
4	Cotton	92.85	93.94	+1.09 ± 1.92	7/3/0
5	Cotton firewood	89.19	91.00	+1.80 ± 2.41	9/1/0
6	Rape	92.48	92.81	+0.33 ± 1.04	5/5/0
7	Chinese cabbage	68.31	70.68	+2.36 ± 3.76	7/3/0
8	Pakchoi	57.29	56.78	-0.51 ± 5.18	5/5/0
9	Cabbage	95.54	96.84	+1.30 ± 1.52	8/2/0
10	Tuber mustard	83.72	85.53	+1.81 ± 4.51	5/5/0
11	Brassica parachinensis	64.88	65.14	+0.26 ± 7.60	6/4/0
12	Brassica chinensis	63.43	64.92	+1.49 ± 9.63	6/4/0
13	Small Brassica chinensis	58.99	62.45	+3.46 ± 6.36	8/2/0
14	Lactuca sativa	86.74	87.84	+1.10 ± 3.17	6/4/0
15	Celtuce	97.29	97.39	+0.10 ± 1.79	6/3/1
16	Film covered lettuce	93.98	94.33	+0.35 ± 2.96	5/5/0
17	Romaine lettuce	90.15	91.11	+0.96 ± 4.94	7/3/0
18	Carrot	94.38	95.05	+0.67 ± 4.34	6/4/0
19	White radish	91.44	93.55	+2.11 ± 2.77	9/1/0
20	Garlic sprout	81.82	82.53	+0.70 ± 0.91	7/3/0
21	Broad bean	89.98	93.36	+3.38 ± 7.67	6/4/0
22	Tree	93.14	93.20	+0.06 ± 5.17	5/5/0

Table S12. WHUHiLongKou Class-Wise Accuracy

Class ID	Class	APMN	BRGC	Δ	+/-/0 seeds
1	Corn	98.44	99.37	+0.93 ± 1.16	8/2/0
2	Cotton	93.04	92.89	-0.15 ± 2.06	5/5/0
3	Sesame	98.22	98.83	+0.61 ± 2.83	5/4/1
4	Broad-leaf soybean	94.47	93.79	-0.68 ± 1.71	3/7/0
5	Narrow-leaf soybean	94.19	96.85	+2.66 ± 4.20	8/2/0
6	Rice	96.83	96.93	+0.10 ± 2.98	5/5/0
7	Water	99.26	99.52	+0.26 ± 0.50	7/3/0

8	Roads and houses	73.35	77.06	+3.71 ± 4.80	7/3/0
9	Mixed weed	83.66	84.79	+1.13 ± 6.09	6/4/0

Table S13. Dataset-Level OA of Matched-Protocol Transductive Baselines

This table reports complete OA results for APMN, standard transductive baselines, and BRGC on 10 target datasets. Graph LP, LaplacianShot, TIM, iLPC, and BRGC use the same APMN base features, the same target 5-shot support sets, and the same unlabeled query sets. They differ only in the transductive calibration mechanism. Values are mean ± std over 10 random seeds. The best and second-best OA values in each row are highlighted in bold and underline, respectively; tied best or tied second-best values are both highlighted.

Dataset	APMN	Graph LP	LaplacianShot	TIM	iLPC	BRGC
IndianPines	76.66 ± 1.81	77.88 ± 1.95	77.88 ± 1.96	68.23 ± 2.21	77.22 ± 1.84	**78.49 ± 1.94**
PaviaU	88.78 ± 3.25	**89.62 ± 3.40**	**89.62 ± 3.40**	78.15 ± 3.35	89.15 ± 3.33	89.34 ± 3.01
Salinas	91.93 ± 1.33	92.20 ± 1.35	92.20 ± 1.35	88.89 ± 1.39	92.03 ± 1.33	**92.36 ± 1.49**
PaviaC	97.99 ± 0.36	98.07 ± 0.35	98.07 ± 0.35	88.14 ± 2.38	98.03 ± 0.35	**98.16 ± 0.40**
Botswana	97.89 ± 1.10	**98.58 ± 1.07**	**98.58 ± 1.06**	97.17 ± 1.15	98.29 ± 1.10	98.53 ± 0.97
KSC	96.42 ± 1.24	**96.84 ± 1.15**	**96.84 ± 1.15**	94.80 ± 1.82	96.62 ± 1.20	96.52 ± 1.67
Houston13	81.05 ± 2.07	81.70 ± 2.21	81.70 ± 2.21	80.28 ± 2.45	81.34 ± 2.14	**82.33 ± 2.15**
WHUHiLongKou	95.83 ± 1.15	95.90 ± 1.15	95.90 ± 1.15	83.80 ± 1.99	95.86 ± 1.15	**96.08 ± 1.19**
WHUHiHanChuan	80.45 ± 1.94	81.13 ± 1.95	81.13 ± 1.95	72.06 ± 3.40	80.72 ± 1.94	**81.17 ± 2.08**
WHUHiHongHu	86.09 ± 1.06	86.95 ± 1.06	86.95 ± 1.06	63.96 ± 4.15	86.42 ± 1.06	**87.34 ± 1.25**
